# Shape‐Memory Metallopolymers Based on Two Orthogonal Metal–Ligand Interactions

**DOI:** 10.1002/adma.202006655

**Published:** 2021-01-14

**Authors:** Josefine Meurer, Julian Hniopek, Thomas Bätz, Stefan Zechel, Marcel Enke, Jürgen Vitz, Michael Schmitt, Jürgen Popp, Martin D. Hager, Ulrich S. Schubert

**Affiliations:** ^1^ Laboratory of Organic and Macromolecular Chemistry (IOMC) Friedrich Schiller University Jena Humboldstr. 10 Jena 07743 Germany; ^2^ Jena Center for Soft Matter (JCSM) Friedrich Schiller University Jena Philosophenweg 7 Jena 07743 Germany; ^3^ Institute of Physical Chemistry (IPC) Friedrich Schiller University Jena Helmholzweg 4 Jena 07743 Germany; ^4^ Abbe Center of Photonics (ACP) Friedrich Schiller University Jena Albert‐Einstein‐Straße 6 Jena 07745 Germany; ^5^ Leibniz Institute of Photonic Technology e. V. Jena, Albert‐Einstein‐Straße 9 Jena 07745 Germany

**Keywords:** metallopolymers, shape‐memory polymers, smart materials, supramolecular polymers

## Abstract

A new shape‐memory polymer is presented, in which both the stable phase as well as the switching unit consist of two different metal complexes. Suitable metal ions, which simultaneously form labile complexes with histidine and stable ones with terpyridine ligands, are identified via isothermal titration calorimetry (ITC) measurements. Different copolymers are synthesized, which contain butyl methacrylate as the main monomer and the metal‐binding ligands in the side chains. Zn(TFMS)_2_ and NiCl_2_ are utilized for the dual crosslinking, resulting in the formation of metallopolymer networks. The switching temperature can simply be tuned by changing the composition as well as by the choice of the metal ion. Strain fixity rates (about 99%) and very high strain recovery rates (up to 95%) are achieved and the mechanism is revealed using different techniques such as Raman spectroscopy.

## Graphical abstract

The synthesis and characterization of shape‐memory metallopolymers based on orthogonal supramolecular interactions are presented; i.e., two different metal complexes. By a cyclo mechanic test, the structure–property relationship is studied, and a strong correlation between the switching temperature, which ranges in this case from 85 to 115 °C, and the structural parameters is achieved.

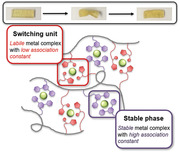

Shape‐memory polymers (SMPs) represent a very interesting class of stimuli‐responsive materials.^[^ ^1^ ^]^ The triggered change from a temporary shape to a preprogrammed permanent one makes them interesting candidates for potential application in, e.g., aerospace^[^ ^2^ ^]^ or biomedicine.^[^ ^3^, ^4^ ^]^

For the general design of a shape‐memory polymer, two different building units are required. First, a stable phase providing strength and functioning as a driving force for the recovery step. Second, the so‐called switching unit, which can be activated by an external trigger.^[^ ^5^, ^6^ ^]^ Both phases can be designed in many different fashions. Typically, chemical (i.e., covalent)^[^ ^7^ ^]^ or physical crosslinking^[^ ^8^ ^]^ are utilized for the formation of the stable phase. The switching unit can be based on different interactions/transitions, like melting point or glass transition temperature (*T*_m_ or *T*_g_)^[^ ^7^, ^9^, ^10^ ^]^ or reversible supramolecular interactions^[^ ^11^, ^12^, ^13^, ^14^, ^15^, ^16^ ^]^ as well as dynamic covalent bonds.^[^ ^17^, ^18^, ^19^, ^20^, ^21^ ^]^

In this study, we present a new shape‐memory polymer, in which not just the switching unit but also the stable phase is consisting of supramolecular metal complexes. Noteworthy, only a single metal salt is required for the dual crosslinking of the two different ligands. Such a system has many advantages in contrast to utilizing a covalent crosslinker for the formation of the stable phase like simple preparation processes and an easy characterization. In our system, first a linear, nicely soluble polymer is synthesized allowing the usage of a wide range characterization techniques (like NMR, size exclusion chromatography (SEC), etc.). Furthermore, the strength of the formed complexes can be tuned by the choice of the metal ion providing a handle for the overall shape‐switching temperature. Last but not least, a slight reversibility of the stable phase provides an easy way for the rewriting of the permanent shape as known for other systems bearing dynamic bonds.^[^ ^22^, ^23^ ^]^

The key parameters for the polymer design are the complex association constants (*K*_α_) of the metal complexes. On the one side a low association constant is required for the switching unit, whereas a very high *K*_α_ is required for the formation of the stable phase. To identify suitable combinations of salts and ligands featuring the desired behavior, isothermal titration calorimetry (ITC) measurements were performed with the 2,2′:6′,2′′‐terpyridine (**Tpy**) ligand and the prepared *N* ^α^‐acetyl‐*N* ^τ^‐trityl‐histidine butyl amide (**His**). The choice of both ligands was based on the fact that terpyridine ligand is known to form strong and stable complexes with certain metal ions (i.e., the ideal candidate for stable phase),^[^ ^24^ ^]^ whereas histidine reveals often only weak interactions with different metal ions (i.e., very well suited for the reversible (switching) phase).^[^ ^25^ ^]^ The results of the ITC study are summarized in Table S1 and Figures S3–S10 in the Supporting Information. Both, zinc(II) trifluoromethane sulfonate (Zn(TFMS)_2_) and nickel(II) chloride, featured the desired complexation behavior, i.e., simultaneous formation of stable terpyridine complexes and labile ones with the histidine ligands. Furthermore, it is important that at the minimum two ligands bind to one metal ion in order to form supramolecular crosslinks. Furthermore, to investigate the procedure of the complex formation when both ligands are present, like it is in the later desired polymers, a 1:1 mixture of both ligands was utilized in the ITC measurement (ITC titration data in Figures S11 and S12 in the Supporting Information). Performing this experiment, it was found that the terpyridine is complexed by half an equivalent of the metal salt in the presence of the histidine. Thus, all the terpyridine ligands are complexed before a complexation of histidine ligands starts and, consequently, no mixed complexes can form, which was additionally confirmed via ^1^H NMR measurements (Figure S13, Supporting Information).

In order to fabricate the dual crosslinked metallopolymer network, linear copolymers containing both ligands as side chains were required. *N* ^α^‐Methacryloyl‐*N* ^τ^‐tritylhistidine butyl amide (**His‐MA**)^[^ ^24^ ^]^ and 6‐(2,2′:6′2′′‐terpyridin‐4′‐yloxy)‐hexyl methacrylate (**Tpy‐MA**)^[^ ^26^ ^]^ were deployed as polymerizable comonomers (for a detailed reaction scheme and the ^1^H NMR spectra see Schemes S1 and S2 and Figures S1 and S2 in the Supporting Information).

Using these two comonomers and *n*‐butyl methacrylate (BMA) as main monomer, four different copolymers (**P1** to **P4**) were synthesized applying the reversible addition fragmentation chain transfer (RAFT) polymerization process (**Figure** **1** and see Table S3 (Supporting Information) for experimental details).^[^ ^27^ ^]^ The amount of the two ligand monomers was varied in order to obtain copolymers with different contents of histidine as switching unit (5% and 10%) and terpyridine (3% and 5%) as basis for the stable phase. All resulting copolymers were characterized using SEC, ^1^H NMR spectroscopy and elemental analysis (EA) (see **Table** **1** and Table S4 and Figures S14–S18 in the Supporting Information). All polymers revealed similar molar masses and, thus, are highly comparable regarding this value. The calculation of the obtained compositions via the ^1^H NMR spectra indicated that the targeted compositions could nearly be achieved.

**Figure 1:**
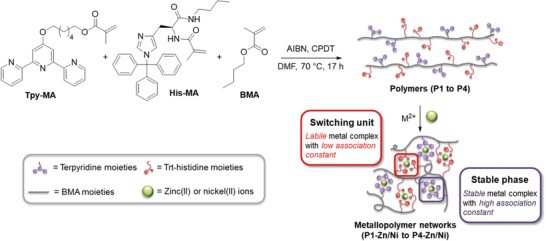
Schematic representation of the synthesis of polymers **P1** to **P4** and the resulting metallopolymer networks **P1‐Zn/Ni** to **P4‐Zn/Ni**.

**Table 1:** Summary of the determined molar masses and dispersities via size‐exclusion chromatography (SEC; CHCl_3_/NEt_3_/*i*‐PrOH (94/4/2), PMMA standard:), calculated composition via ^1^H NMR spectroscopy and thermal properties (determined glass transition temperature via differential scanning calorimetry (DSC) and degradation temperature via thermo gravimetric analysis (TGA))

	SEC results^a)^			Calc. composition via ^1^H NMR^b)^		Thermal properties		
	*M*_n_[g mol^−1^]	*M*_w_[g mol^−1^]	*Ð*	His‐Units[%]	Tpy‐Units[%]	*T*_g_[°C]	*T*_d_ ^c)^[°C]	*T*_d_ ^d)^[°C]
**P1**	18 000	22 00	1.19	11.0	4.0	34	199	250
**P2**	16 700	20 100	1.20	5.4	4.0	28	250	248
**P3**	18 100	21 400	1.18	12.5	3.0	34	198	252
**P4**	16 900	20 100	1.18	5.4	3.0	25	184	246

^a)^ Eluent: CHCl_3_/NEt_3_/*i*‐PrOH (94/4/2), PMMA standard

^b)^ 300 MHz, CD_2_Cl_2_

^c)^ heating rate: 5 K min^−1^

^d)^ heating rate: 20 K min^−1^.

Subsequently, the metallopolymer networks were obtained by simple addition of the corresponding metal salt to the dissolved polymers (see Table S5 in the Supporting Information). Zinc(II) trifluoromethane sulfonate was utilized to synthesize the metallopolymer networks **P1‐Zn** to **P4‐Zn** and nickel(II) chloride hexahydrate for metallopolymer networks **P1‐Ni** to **P4‐Ni** (Figure 1).

To proof the successful formation of the histidine and the terpyridine complexes within the polymer networks, Raman spectroscopy was applied. Model complexes, containing **Tpy** or **His** and the respective metal salt (**[(His)_2_Zn]^2+^**, **[(His)_3_Ni]^2+^**, **[(Tpy)_2_Zn]^2+^**, and **[(Tpy)_2_Ni]^2+^**), were synthesized for comparison and to allow an assignment of the bands (see Table S2 and Scheme S3 in the Supporting Information).

The results from the model systems could subsequently be utilized to analyze the changes in the Raman spectra of the metallopolymers (spectra are included in Figures S36–S40 in the Supporting Information). Since the spectra of the polymers reveal overlapping features of both the **His** and the **Tpy** ligand, the changes are subtler compared to the respective model systems: The shift at 996 cm^−1^ originating from the **Tpy** moiety is only visible by the appearance of a shoulder at 1020 cm^−1^, since it is overlaid by strong vibrations from the **His**. The same holds true for the shift at 1566 cm^−1^ originating from the **His** ligand, which coincides with the changes in the **Tpy** ligand. In spite of those overlaps, clear indications for a coordination of the respective metal ions to both ligand moieties can be found in the Raman spectra at 1352 and 1595 cm^−1^, where no overlapping features complicate the analysis. The Raman‐spectroscopic measurements were therefore able to confirm a successful complexation of both Ni^2+^ and Zn^2+^ ions to the **His** as well as the **Tpy** ligands in the model systems and all metallopolymers.

Furthermore, the thermal properties of all polymers and metallopolymer networks were investigated via differential scanning calorimetry (DSC) and thermogravimetric analysis (TGA) (Table S6 and Figures S19–S26, Supporting Information). The DSC measurements revealed that all polymers show a glass transition temperature (*T*_g_) around 30 °C (see Table 1), which was expected for these BMA containing linear copolymers.^[^ ^28^ ^]^ In contrast, the metallopolymer networks showed a much higher glass transition temperature. This shift is presumably due to the high supramolecular crosslinking density. The presence of both, the stable and the labile ligand could be the origin of the broadening. The TGA measurements confirmed the good thermal stability of all materials (degradation about 180 to 250 °C).

In order to study the shape‐memory effect for all metallopolymer networks (**P1‐Zn/Ni** to **P4‐Zn/Ni**) a permanent shape had to be determined first. For this propose, these networks were pressed at 140 to 150 °C in a rectangular shape (see (A) in **Figure** **2**a) with the help of a manufactured mold and a pressure of about 1.5 tons. Subsequently, the rectangular polymer pieces were heated to 120 °C, or in case of **P1‐Zn/Ni** and **P3‐Zn** to 130 °C, twisted at this elevated temperature and were subsequently cooled immediately to room temperature to fix the temporary shape (see (B) in Figure 2a). Afterwards, the polymers were heated again from room temperature to 120 and 130 °C, respectively. After ≈15 min the permanent rectangular shape was almost completely recovered (see (C) in Figure 2a). For the metallopolymer networks, which showed the best performance in this shape‐memory test, the procedure was repeated twice (see (D–G) in Figure 2a). A photo series of this shape‐memory test is shown in Figure 2a, the photo series of the metallopolymer networks **P1‐Ni**, **P3‐Zn**, and **P4‐Ni** are shown in Figure S41 in the Supporting Information. A deterioration during the cycles could be observed for none of the multiple tested metallopolymer networks. It has to be noted that the temperatures, at which this manual shape‐memory test was performed, are not the real switching temperatures. Due to the heat loss during operation (opening the oven, taking out the sample, twisting the sample) the temperature had to be chosen slightly higher. Nevertheless, these first tests indicated that the metallopolymer networks **P1‐Zn** and **P1‐Ni**, which contained the highest proportion of both ligands, revealed a higher switching temperature and were stiffer compared to the metallopolymer networks with the lowest content of both ligands (**P4‐Zn** and **P4‐Ni**), which were at the lower temperature already relative soft and the easiest to deform.

**Figure 2:**
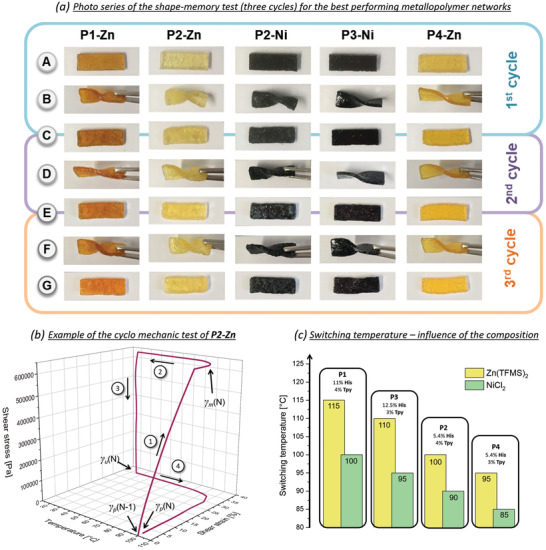
a) Photo series of the shape‐memory test of the metallopolymer networks which revealed the best performance (programming and recovery at: 120 °C for **P2‐Zn/Ni**, **P3‐Ni**, and **P4‐Zn**; 130 °C for **P1‐Zn**). (A) Permanent shape 1st cycle; (B) temporary shape 1st cycle; (C) recovered permanent shape 1st cycle and new permanent shape 2nd cycle; (D) temporary shape 2nd cycle, (E) recovered permanent shape 2nd cycle and new permanent shape 3rd cycle; (F) temporary shape 3rd cycle; (G) recovered permanent shape 3rd cycle. b) Temperature–stress–strain diagram of the metallopolymer network **P2‐Zn** (second cycle); (1) deformation at 95 °C; (2) cooling to 25 °C under constant stress; (3) releasing the stress at 25 °C; (4) heating to 95 °C and recovery of the permanent shape; c) correlation of the switching temperatures of the metallopolymer networks.

Cyclo mechanic tests were performed to investigate the shape memory abilities of the metallopolymer networks in more detail. This test can be used to determine the strain fixity rate (*R*_f_), which quantifies the ability to fix the temporary shape (γ_u_) after mechanical deformation (γ_m_). Furthermore, it is possible to quantify the ability to restore the mechanical deformation and with this the recovery of the permanent shape (γ_p_), the so‐called strain recovery rate (*R*_r_). The test was repeated four times (*N* = 1–4) for each sample. Equations (1) and (2) were used to calculate the values for the fixity and recovery rates of the metallopolymer networks **P1‐Zn/Ni** to **P4‐Zn/Ni**, which are summarized in **Table** **2** (and Table S7 in the Supporting Information).

(1) $$\begin{array}{c} R_{f}=\;\frac{\gamma_{u}\left(N\right)}{\gamma_{m}\left(N\right)}\times \;100 \end{array}$$

(2) $$\begin{array}{c} R_{r}=\;\frac{\gamma_{m}\left(N\right)-\gamma_{p}\left(N\right)}{\gamma_{m}\left(N\right)-\gamma_{p}\left(N-1\right)}\times \;100 \end{array}$$

**Table 2:** Ideal switching temperatures and calculated strain fixity as well as recovery rates (for the cycles 1–4) of the metallopolymer networks **P1‐Zn/Ni** to **P4‐Zn/Ni**

Sample	*T*_sw_ [°C]	*R*_f_ [%]				*R*_r_ [%]			
		Cycle				Cycle			
		1	2	3	4	1	2	3	4
**P1‐Zn**	115	99.7	99.7	99.6	99.6	77.5	85.6	86.1	87.1
**P1‐Ni**	100	99.6	99.0	99.0	99.3	71.3	71.6	65.8	69.1
**P2‐Zn**	100	99.8	99.7	99.7	99.8	86.4	94.4	95.3	95.3
**P2‐Ni**	90	99.5	99.7	99.7	99.7	77.6	84.1	85.8	86.5
**P3‐Zn**	110	99.6	99.5	99.4	99.4	70.0	81.1	80.7	81.4
**P3‐Ni**	95	100	99.6	100	100	63.9	77.3	80.6	81.1
**P4‐Zn**	95	99.0	98.8	98.8	99.2	86.0	90.9	91.3	90.8
**P4‐Ni**	85	98.4	98.2	97.5	97.7	78.0	78.7	76.5	76.6

The ideal switching temperature was found to be the lowest temperature, at which it was still possible to deform the sample without breaking (tested in Δ*T* = 5 °C steps). Higher switching temperatures resulted in all cases in worse recovery rates, which is presumably due to a partial rewriting of the permanent shape caused by the activation of the slightly addressable terpyridine complexes. An example of this phenomenon is depicted in Figure S35 in the Supporting Information.

During the cyclo‐mechanic test at the ideal switching temperature, all metallopolymer networks showed excellent strain fixity ratios from 98.2% to 99.8% and also the calculated strain recovery ratios revealed high values up to 95.4%. These values are comparable to already existing metallopolymer systems.^[^ ^14^, ^15^, ^16^, ^29^ ^]^ It has to be noticed that during nearly all measurements the first cycle revealed a smaller recovery rate compared to the following three cycles. Such a phenomenon is already known from other shape‐memory polymers and it probably results from the thermal history as well as potential remaining stress in the sample due to the fabrication process.^[^ ^30^ ^]^

With this test, we could determine the trend that metallopolymer networks containing zinc(II)‐ions showed better recovery rates compared to the nickel(II)‐ion containing ones, both being prepared from the same polymer. Polymer networks **P2‐Zn** and **P4‐Zn** revealed the best performance indicating that a lower histidine content is beneficial.

Exemplarily, the 3D‐plot of the second cycle for the metallopolymer network **P2‐Zn** is displayed in Figure 2b. The plots of all cycles for all metallopolymer networks are depicted in Figures S27–S34 in the Supporting Information.

The ideal switching temperatures range from 85 to 115 °C, depending on the polymer composition and the corresponding metal salt. A comparison for all metallopolymer networks is shown in Figure 2c and the following trends could be identified: First, all nickel(II)‐containing metallopolymer networks featured a much lower switching temperatures. This behavior is probably associated on the one hand with a stronger binding behavior of the histidine to the zinc (see ITC‐results) and on the other hand with the stoichiometries of the formed histidine complex. As a consequence, a strong correlation between the ITC‐results (binding constant and stoichiometry) and the shape‐memory behavior could be obtained.

Second, the metallopolymer networks featuring an overall higher ligand content **P1** and **P3** showed a higher switching temperature compared to the networks with the overall lower content **P2** and **P4**. This finding is valid for both metal ions used. Third, a higher terpyridine content lead to higher temperatures as can be seen from the comparisons of **P1** versus **P3** as well as of **P2** versus **P4**.

Overall, a strong correlation between the structural parameters (binding constant, the stoichiometry of the metal–ligand interaction and the content of ligand) and the resulting property (shape‐memory) could be revealed. Thus, a defined molecular plan of a polymer would enable a tailor‐made design of metallopolymers with a predefined shape‐memory temperature.

In summary, we have realized novel shape‐memory polymers by the combination of two supramolecular interactions—namely metal complexes of terpyridine as well as histidine—which show excellent strain fixity rates about 99% and strain recovery rates up to 95%. Our approach combines the formation of strong terpyridine complexes as the stable phase with the formation of labile histidine complexes building up the switching units based on the simultaneous complexation of linear copolymers in a single step. Consequently, a straight‐forward and simple access to these materials is possible. By the combination of NMR and Raman spectroscopy as well as ITC measurements the complexation with zinc(II)‐ and nickel(II)‐ions could be investigated in detail. A systematic library of metallopolymer networks was synthesized, in which the overall ligand content, the content of the stable phase and the nature of the metal ion were varied. These parameters can be utilized to modulate the switching temperature between 85 and 115 °C. Hereby, zinc(II)‐ions, a higher overall ligand content and a higher terpyridine content result in higher switching temperatures. The stability of these materials makes them usable for multiple cycles. These new shape‐memory metallopolymers will enable the design of tailor‐made SMPs.

Within this context, follow‐up studies will cover the tuning of the metallopolymer system by the variation of the ligands and/or the used metal salts for the formation of the complexes (e.g., the use of different salts for both ligands). Furthermore, the observed rewriting of the permanent shape at elevated temperatures will be investigated in detail.

## Experimental Section

Detailed information about the synthesis and characterization of the monomers, model compounds, polymers, and metallopolymer networks are provided in the Supporting Information.

## Conflict of Interest

The authors declare no conflict of interest.

## Author Contribution

Synthesis of monomers and polymers: J.M.; SEC, DSC, TGA, and shape‐memory tests: J.M.; Raman measurements: J.H.; DFT calculations: J.H.; ITC‐measurements: M.E., T.B.; rheology measurements: J.M., J.V., writing of the manuscript: J.M, J.H., and S.Z.; interpretation of measurement data: J.M., J.H., S.Z., supervision: S.Z., M.D.H., U.S.S., M.S., J.P.; concept of the study: S.Z., M.D.H., U.S.S., M.S., J.P; correction of the manuscript: M.D.H., U.S.S., M.S., J.P.

## Supporting information

Supporting Information
