# Supplementary material for: Shape‐Memory Metallopolymers Based on Two Orthogonal Metal–Ligand Interactions
Source: Adv Mater. 2021 Jan 14;33(7):2006655. doi: 10.1002/adma.202006655 (PMC11469176; doi:10.1002/adma.202006655)
Supplement: Supplementary file 1 — Supporting Information [file ADMA-33-2006655-s001.pdf]

© 2021 Wiley-VCH GmbH

ADVANCED MATERIALS

Supporting Information

for *Adv. Mater.*, DOI: 10.1002/adma.202006655

Shape-Memory Metallopolymers Based on Two Orthogonal Metal–Ligand Interactions

*Josefine Meurer, Julian Hniopek, Thomas Bätz, Stefan Zechel, Marcel Enke, Jürgen Vitz, Michael Schmitt, Jürgen Popp, Martin D. Hager, and Ulrich S. Schubert**

Supporting Information

Shape-Memory Metallopolymers Based on Two Orthogonal Metal–Ligand Interactions

Josefine Meurer, Julian Hniopek, Thomas Bätz, Stefan Zechel, Marcel Enke, Jürgen Vitz, Michael Schmitt, Jürgen Popp, Martin D. Hager and Ulrich S. Schubert,*

J. Meurer, T. Bätz, Dr. S. Zechel, Dr. M. Enke, Dr. J. Vitz, Dr. M. D. Hager, Prof. U. S. Schubert
Laboratory of Organic and Macromolecular Chemistry (IOMC), Friedrich Schiller University Jena, Humboldstr. 10, 07743 Jena, Germany, E-mail: ulrich.schubert@uni-jena.de

J. Meurer, T. Bätz, Dr. S. Zechel, Dr. M. Enke, Dr. J. Vitz, Prof. J. Popp, Dr. M. D. Hager, Prof. U. S. Schubert
Jena Center for Soft Matter (JCSM), Friedrich Schiller University Jena, Philosophenweg 7, 07743 Jena, Germany

J. Hniopek, Prof. M. Schmitt, Prof. J. Popp
Institute of Physical Chemistry (IPC), Friedrich Schiller University Jena, Helmholzweg 4, 07743 Jena, Germany

J. Hniopek, Prof. M. Schmitt, Prof. J. Popp
Abbe Center of Photonics (ACP), Friedrich Schiller University Jena, Albert-Einstein-Straße 6, 07745 Jena, Germany

J. Hniopek, Prof. J. Popp
Leibniz Institute of Photonic Technology, e. V. Jena, Albert-Einstein-Straße 9, 07745 Jena, Germany

Table of contents

Materials and methods 3

Synthesis of the histidine monomers (**His-MA**) and the model complex (**His**) 4

NMR of N^{τ}-tritylhistidine butyl amide (**3**) 6

NMR of N^{α}-acetyl-N^{τ}-tritylhistidine butyl amide (**His**) 6

NMR of N^{α}-methacryloyl-N^{τ}-tritylhistidine butyl amide (**His-MA**) 6

Synthesis of the terpyridine monomers (**Tpy-MA**) 7

NMR of 6-(2,2':6'2''-terpyridin-4'-yloxy)-hexan-1-ol (**5**) 9

NMR of 6-(2,2':6'2''-terpyridin-4'-yloxy)-hexyl methacrylate (**Tpy-MA**) 9

Isothermal titration calorimetry (ITC) 10

Synthesis of the model complexes 16

Proof of the selective complex formation *via* ^{1}H NMR spectroscopy 18

Synthesis of the polymers **P1** to **P4** *via* RAFT polymerization 19

Size exclusion chromatography (SEC) 22

Synthesis of the metallopolymer networks 24

Differential scanning calorimetry (DSC) 26

Thermo gravimetric analysis (TGA) 28

Cyclo-mechanic tests 30

Comparison of the R_r depending on the temperature (exemplarily for **P2-Ni**) 40

Raman-spectroscopic measurements 41

Density functional theory calculations 43

Band assignments for **Tpy** and **His** 44

Raman-spectra of polymer(s) (networks) 46

Additional photo series of the shape-memory test 48

References 49

Materials and methods

All chemicals were used as received from TCI (Eschborn, Germany), Sigma Aldrich (Darmstadt, Germany), Alfa Aesar (Kandel, Germany), Thermo Fisher Scientific (Geel, Belgium) and Acros Organics (Geel, Belgium) if not otherwise stated. All solvents were dried over molecular sieve under nitrogen atmosphere. The stabilizer in the used liquid monomer butyl methacrylate was removed over a short aluminium oxide (AlOx) column (neutral AlOx, obtained from Molecula, Darlington, UK).

Nuclear magnetic resonance spectra were measured using a Bruker AC 250 (250 MHz), Bruker AC 300 (300 MHz), Bruker AC 400 (400 MHz) and a Bruker AC 600 (600 MHz) spectrometers (Billerica, MA, USA) at 298 K if not stated differently. The chemical shift is given in parts per million (ppm on δ Scale) related to deuterated solvent.

Elemental analysis was performed utilizing a Vario El III (Elementar, Langenselbold, Germany).

Synthesis of the histidine monomers (**His-MA**) and the model complex (**His**)

The histidine monomer (**His-MA**) and the model complex (**His**) were synthesized according to a literature producer.[1] The schematic representation of the synthesis is shown in **Figure S1**. The ^{1}H NMR spectra are displayed in **Figure S2**.

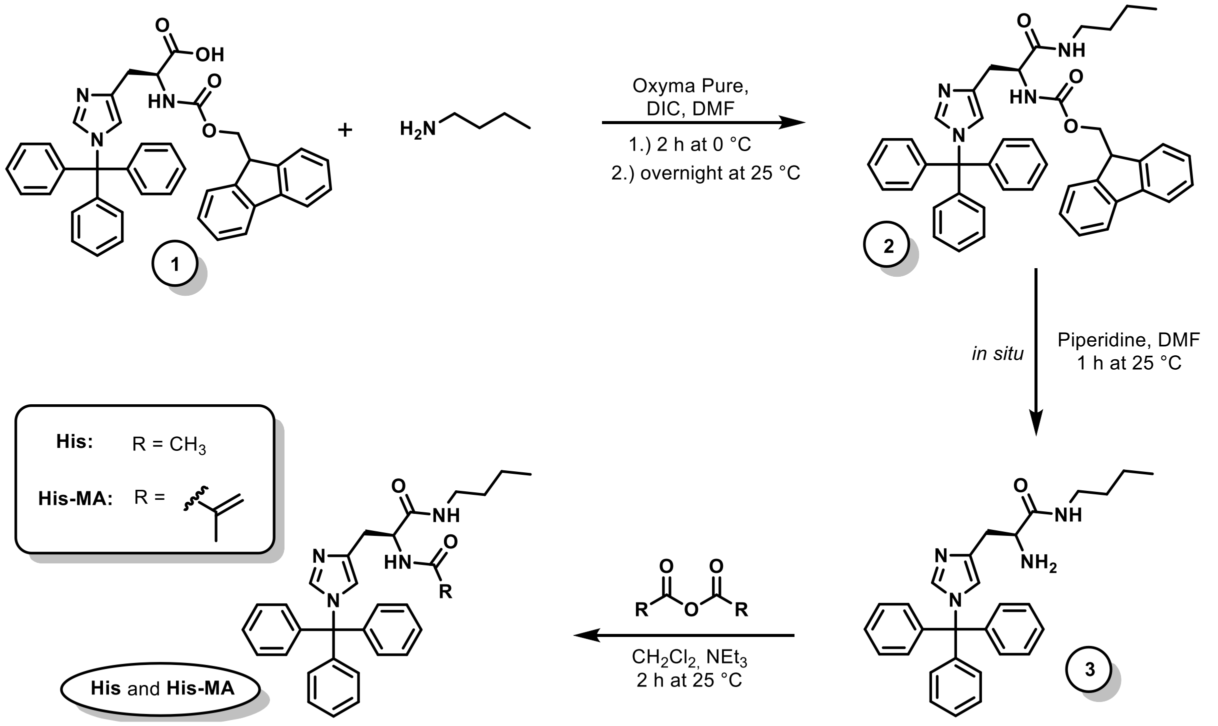

Scheme S1: Schematic representation of the synthesis of the histidine monomer (**His-MA**) and the model system (**His**). DIC = *N,N'*-diisopropyl carbodiimide; DMF = *N,N'*-dimethyl formamide.

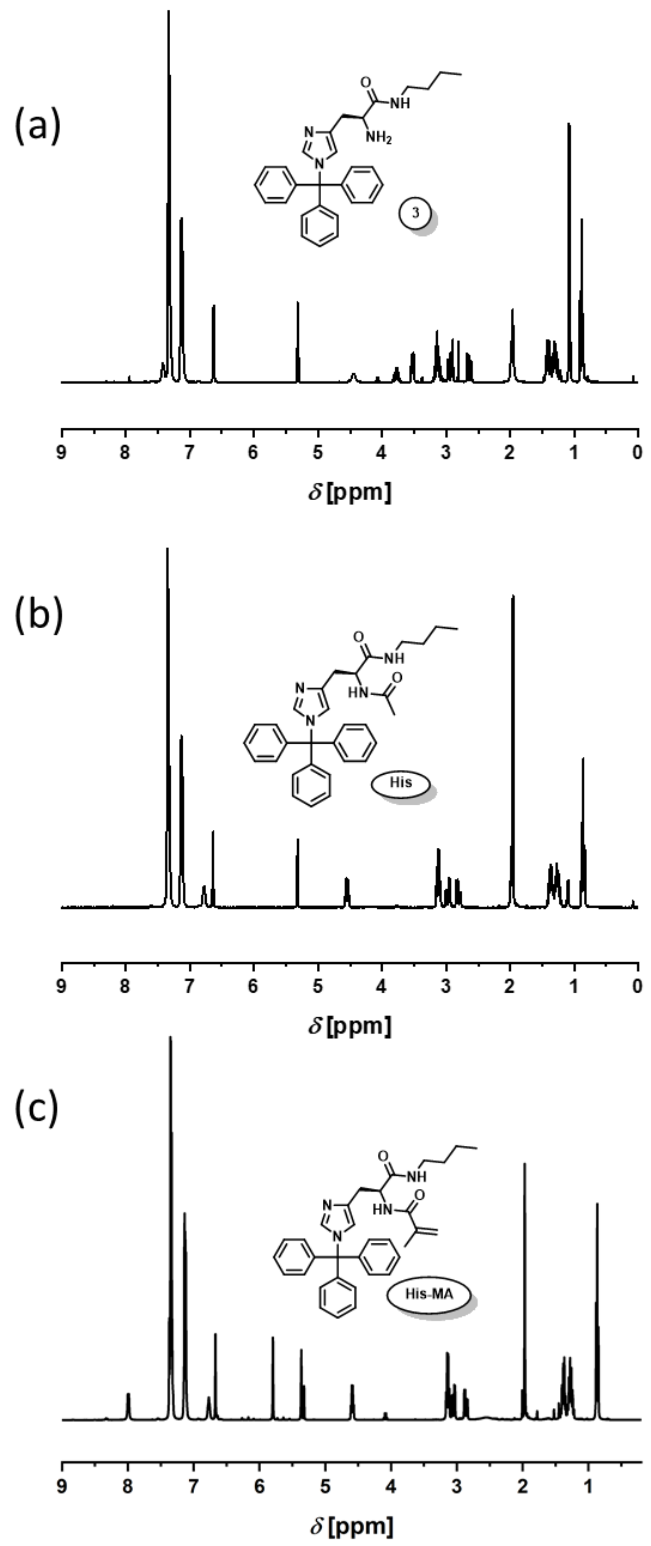

Figure S1: ^1H NMR spectra of: (a) Compound **3**, (b) the histidine model system (**His**) and (c) the histidine monomer (**His-MA**) (300 MHz, CD_2Cl_2).

NMR of N^{τ}-tritylhistidine butyl amide (**3**)

^{1}H NMR (300 MHz, CD_2Cl_2): δ = 7.42 (s, 1H), 7.34–7.29 (m, 10H), 7.14–7.11 (m, 6H), 6.63 (s, 1H), 3.54–3.50 (m, 1H), 3.16–2.60 (m, 4H), 1.98 (s, 2H), 1.47–1.20 (m 4H), 0.91–0.86 (t, 3H) ppm.

NMR of N^{α}-acetyl-N^{τ}-tritylhistidine butyl amide (**His**)

^{1}H NMR (300 MHz, CD_2Cl_2): δ = 7.53–7.24 (m, 10H), 7.16 (dd, J = 6.6, 2.9 Hz, 6H), 6.81 (s, 1H), 6.67 (s, 1H), 4.58 (dd, J = 12.2, 6.5 Hz, 1H), 3.31–2.64 (m, 4H), 2.00 (d, J = 8.6 Hz, 4H), 1.54–1.03 (m, 5H), 0.89 (t, J = 7.2 Hz, 3H) ppm.

NMR of N^{α}-methacryloyl-N^{τ}-tritylhistidine butyl amide (**His-MA**)

^{1}H NMR (300 MHz, CD_2Cl_2): δ = 7.99 (d, J = 6.5 Hz, 1H), 7.37–7.34 (m, 10H), 7.13 (dd, J = 6.4, 3.3 Hz, 6H), 6.67 (s, 1H), 5.80 (s, 1H), 5.36 (s, 1H), 4.61–4.57 (m, 1H), 3.20 – 2.79 (m, 4H), 1.97 (s, 3H), 1.47 – 1.16 (m, 5H), 0.87 (t, J = 7.3 Hz, 3H) ppm.

Synthesis of the terpyridine monomers (**Tpy-MA**)

The terpyridine monomer (**Tpy-MA**) was synthesized according to literature.[2] The schematic representation of the synthesis is shown in **Figure S3**. The ^{1}H spectra are displayed in **Figure S4**.

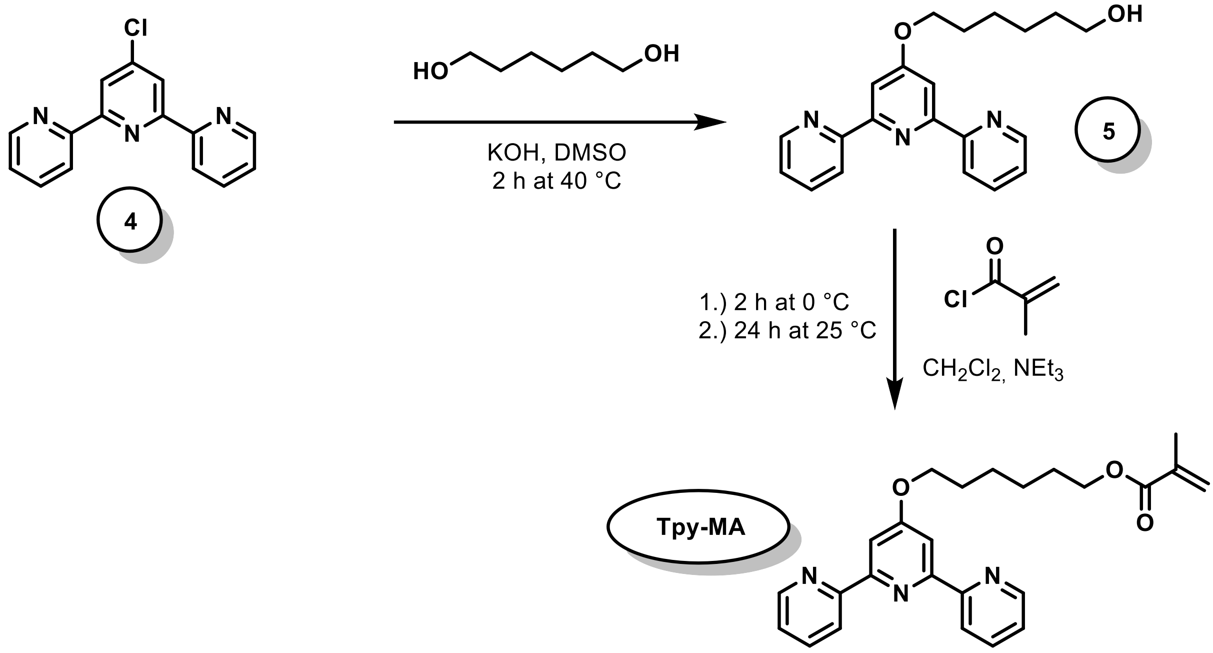

Scheme S2: Schematic representation of the synthesis of the terpyridine monomer (**Tpy-MA**).

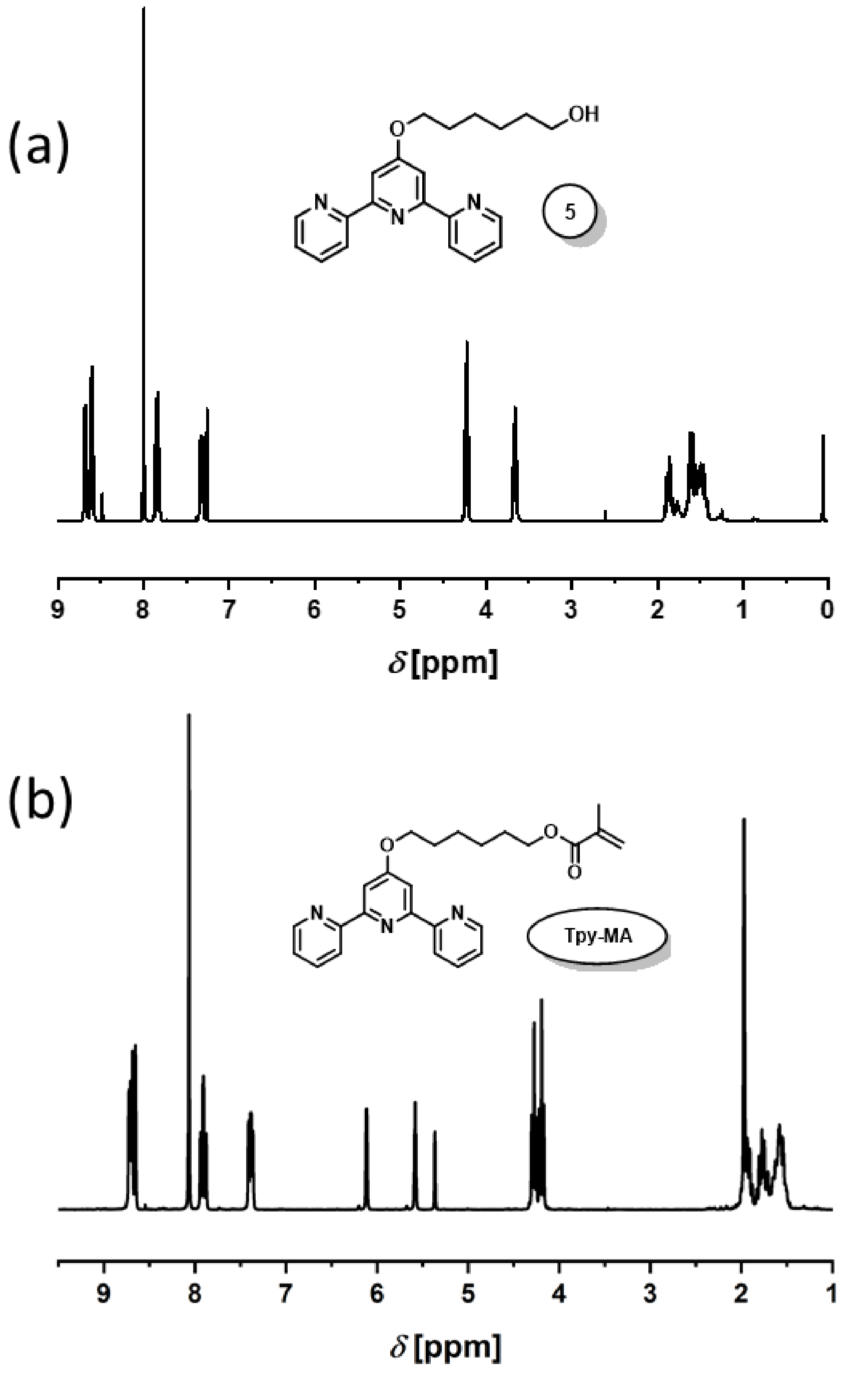

Figure S2: ^{1}H NMR spectra of (a) compound **5** (300 MHz, $CDCl_3$) and (b) the terpyridine monomer (**Tpy-MA**) (300 MHz, CD_2Cl_2).

NMR of 6-(2,2’:6’2’’-terpyridin-4’-yloxy)-hexan-1-ol (**5**)

^{1}H NMR (300 MHz, $CDCl_3$): δ = 8.74–8.43 (m, 4H), 7.92 (s, 2H), 7.83–7.67 (m, 2H), 7.40–7.06 (m, 2H), 4.14 (t, J = 6.4 Hz, 2H), 3.58 (t, J = 5.7 Hz, 2H), 1.97–1.11 (m, 9H) ppm.

NMR of 6-(2,2’:6’2’’-terpyridin-4’-yloxy)-hexyl methacrylate (**Tpy-MA**)

^{1}H NMR (300 MHz, CD_2Cl_2): δ = 8.78–8.60 (m, 4H), 8.05 (s, 2H), 7.98–7.81 (m, 2H), 7.37 (ddd, J = 7.5, 4.8, 1.2 Hz, 2H), 6.10 (s, 1H), 5.56 (s, 1H), 4.22 (dt, J = 26.4, 6.5 Hz, 4H), 2.02–1.40 (m, 11H) ppm.

Isothermal titration calorimetry (ITC)

All titrations were performed using a standard volume Nano ITC (TA Instruments) at 303 K. The solutions were always prepared prior to use in dry solvents utilizing vacuum dried ligand and metal salt. Blank titrations in dry solvent were performed and subtracted from the corresponding titrations to remove the effect of dilution. The fitting of the measured data was performed with the NanoAnalyze program from TA instruments.

Table S1. Determined complex association constants (K_α) and stoichiometry (n) for **Tpy** or **His** and different metal salts by ITC measurements.

**Metal salt**	**Terpyridine (Tpy)**		**Histidine (His)**	
	**K_α**	**n**	**K_α**	**n**
$Zn(OAc)_2$	2.19×10^5	1.07	1.78×10^2	3.17
$ZnCl_2$	2.90×10^5	2.23	3.87×10^2	2.14
$Zn(TFMS)_2$	3.24×10^6	1.90	6.02×10^2	2.23
$NiCl_2$	9.98×10^9	1.69	2.84×10^2	3.71

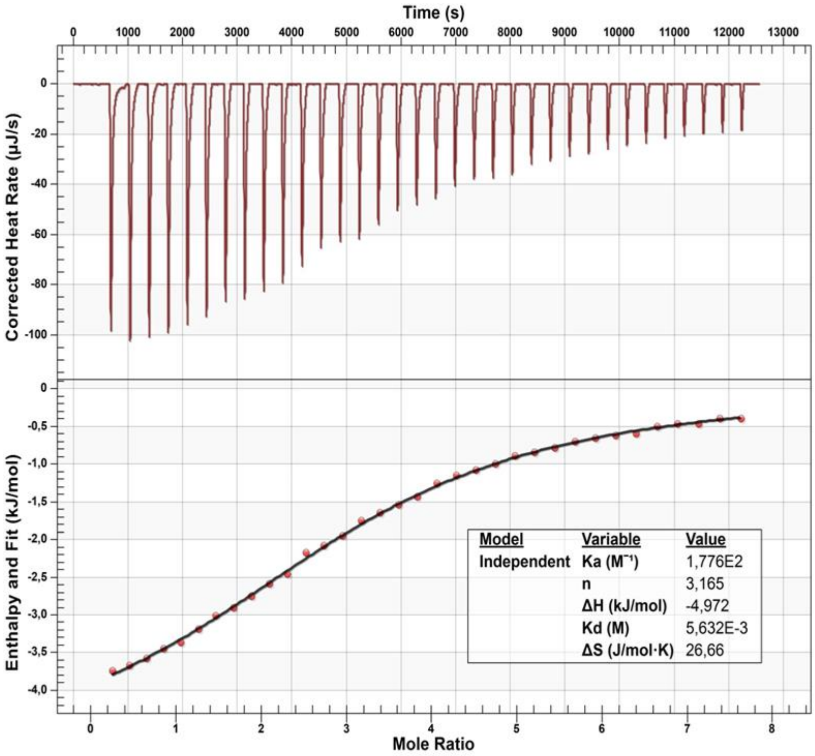

Figure S3: ITC titration data of $Zn(OAc)_2$ (6.24 mM, in cell) with **His** (169.8 mM, in syringe) in MeOH/$CHCl_3$ (2:1) at 303 K.

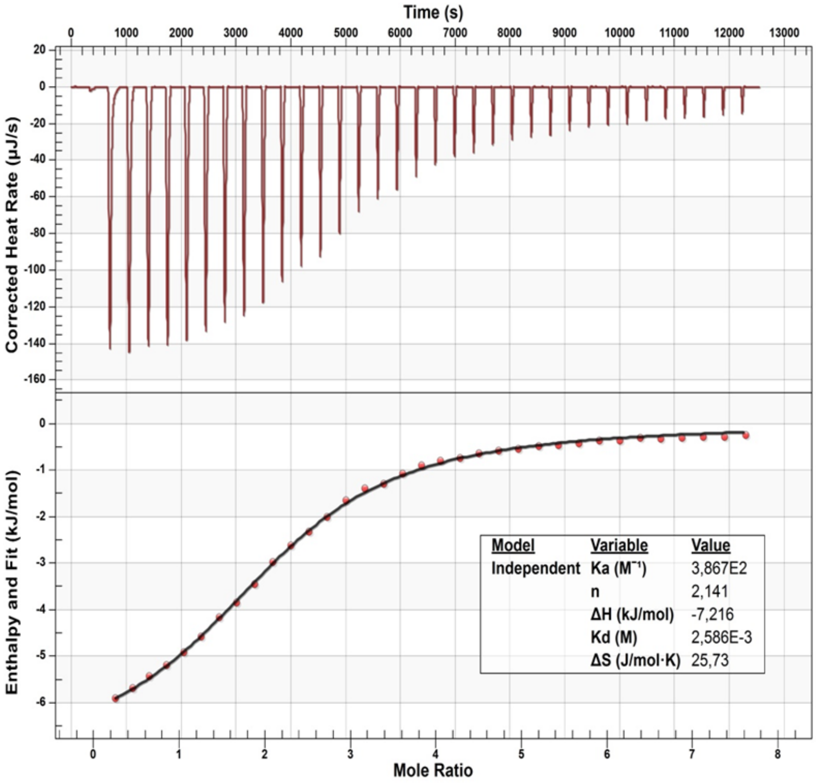

Figure S4: ITC titration data of $ZnCl_2$ (6.25 mM, in cell) with **His** (171.10 mM, in syringe) in MeOH/$CHCl_3$ (2:1) at 303 K.

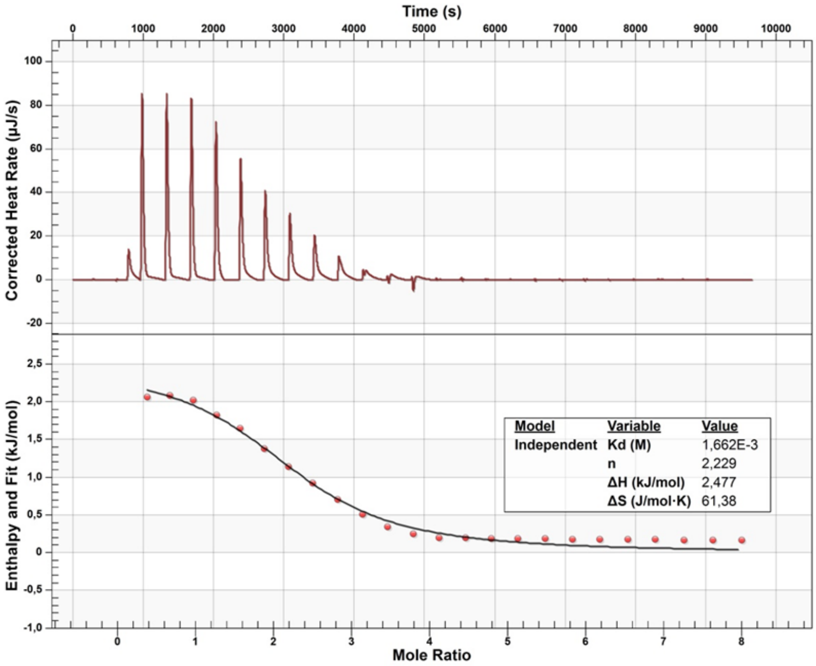

Figure S5: ITC titration data of Zn(TFMS)$_2$ (6.23 mM, in cell) with **His** (171.10 mM, in syringe) in MeOH/CHCl$_3$ (2:1) at 303 K.

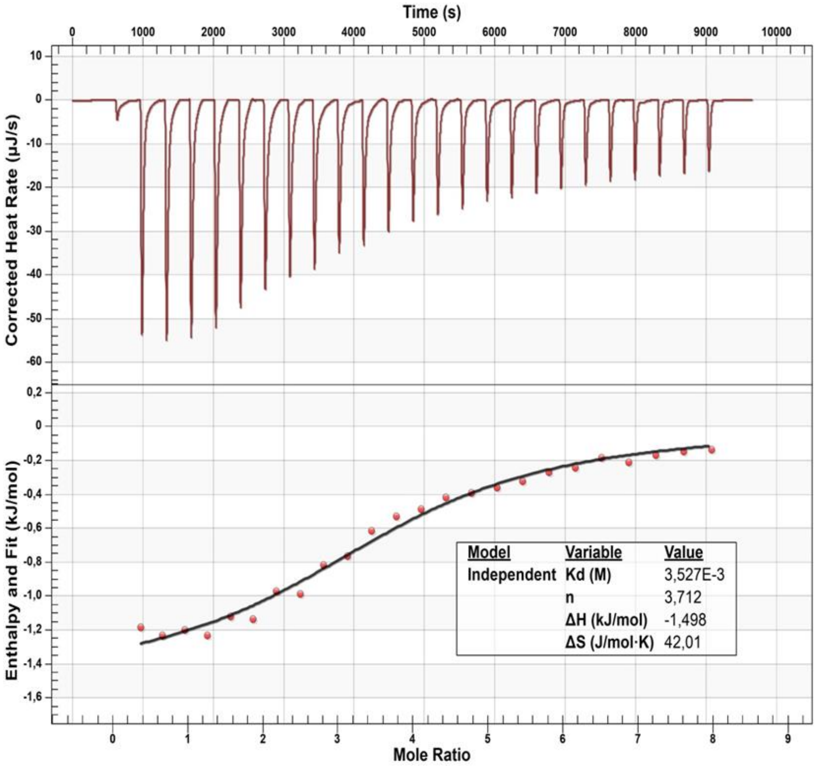

Figure S6: ITC titration data of NiCl$_2$ (6.25 mM, in cell) with **His** (171.10 mM, in syringe) in MeOH/CHCl$_3$ (2:1) at 303 K.

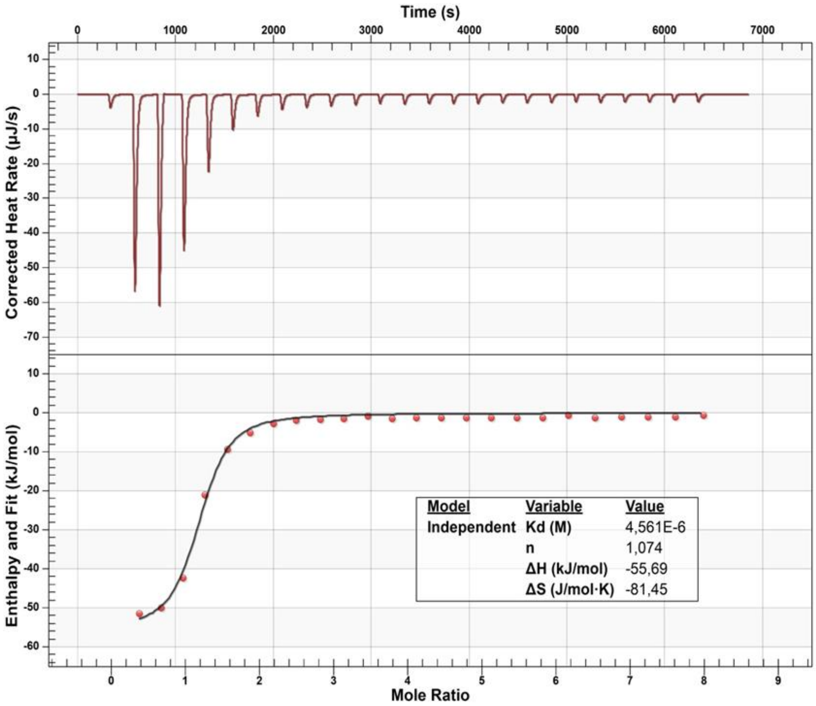

Figure S7: ITC titration data of $Zn(OAc)_2$ (0.12 mM, in cell) with **Tpy** (3.43 mM, in syringe) in MeOH/$CHCl_3$ (2:1) at 303 K.

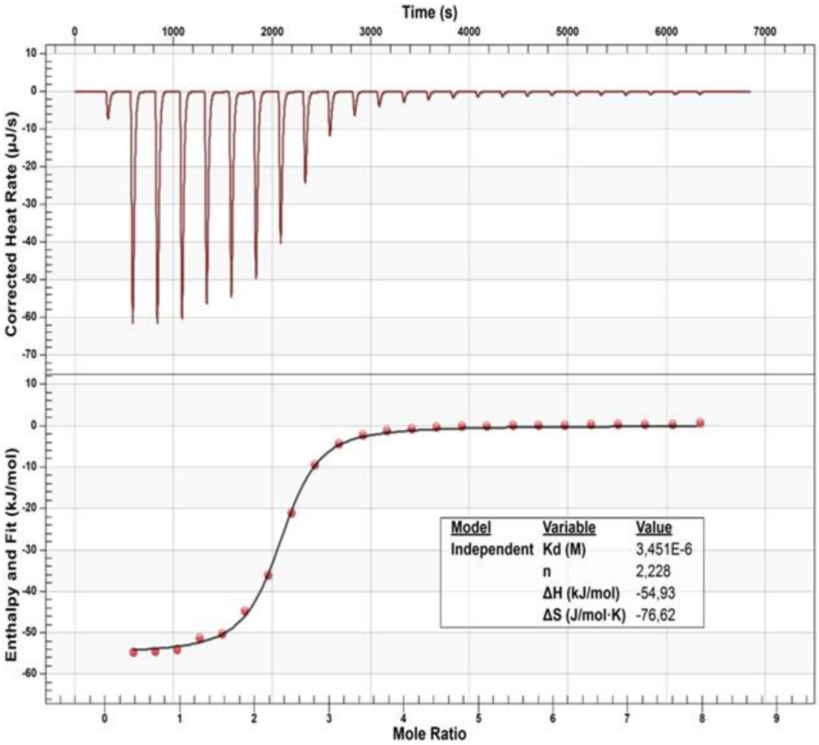

Figure S8: ITC titration data of $ZnCl_2$ (0.13 mM, in cell) with **Tpy** (3.42 mM, in syringe) in MeOH/$CHCl_3$ (2:1) at 303 K.

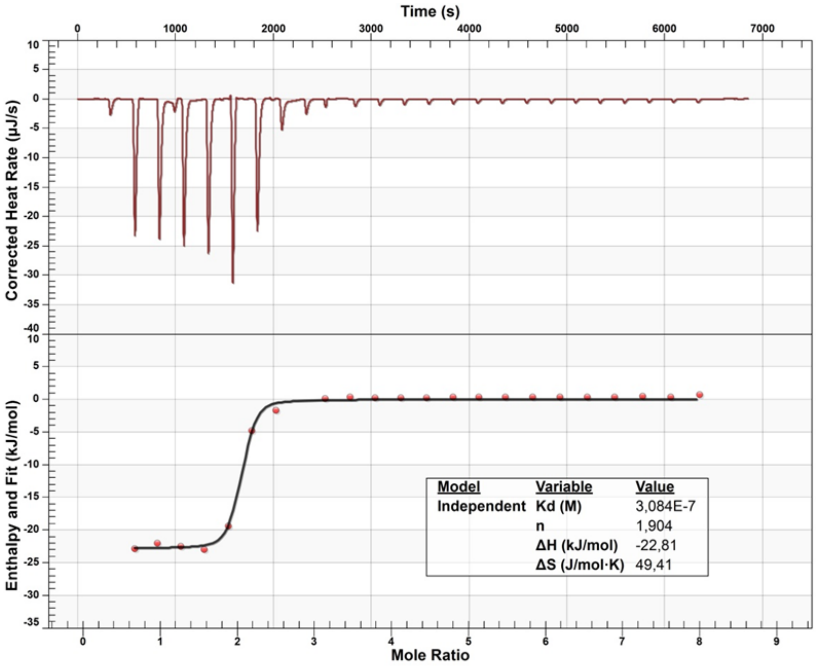

Figure S9: ITC titration data of $Zn(TFMS)_2$ (0.12 mM, in cell) with **Tpy** (3.43 mM, in syringe) in $MeOH/CHCl_3$ (2:1) at 303 K.

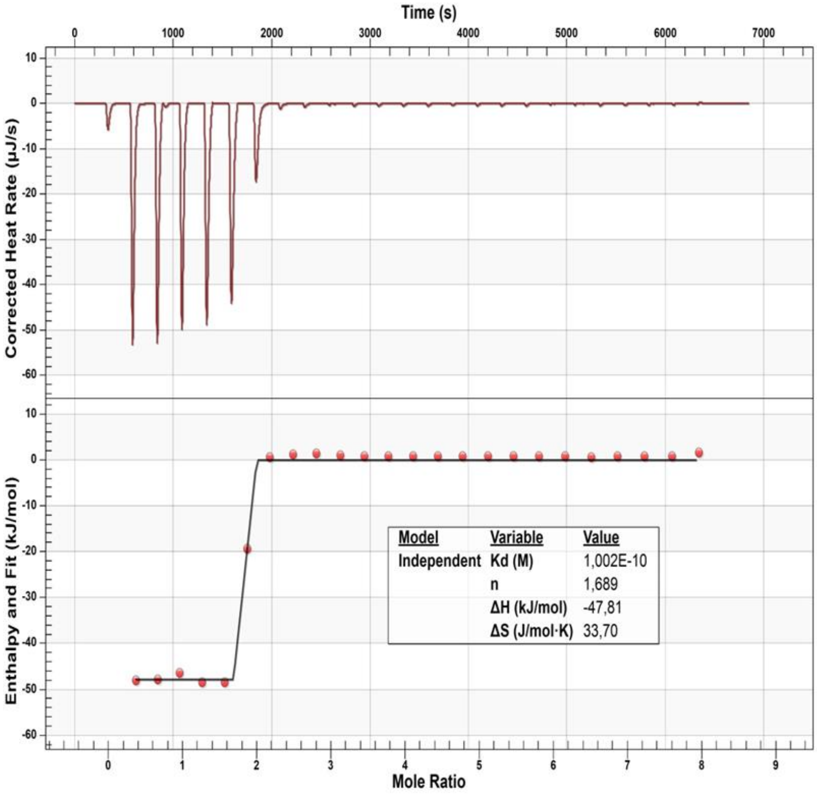

Figure S10: ITC titration data of $NiCl_2$ (0.13 mM, in cell) with **Tpy** (3.42 mM, in syringe) in $MeOH/CHCl_3$ (2:1) at 303 K.

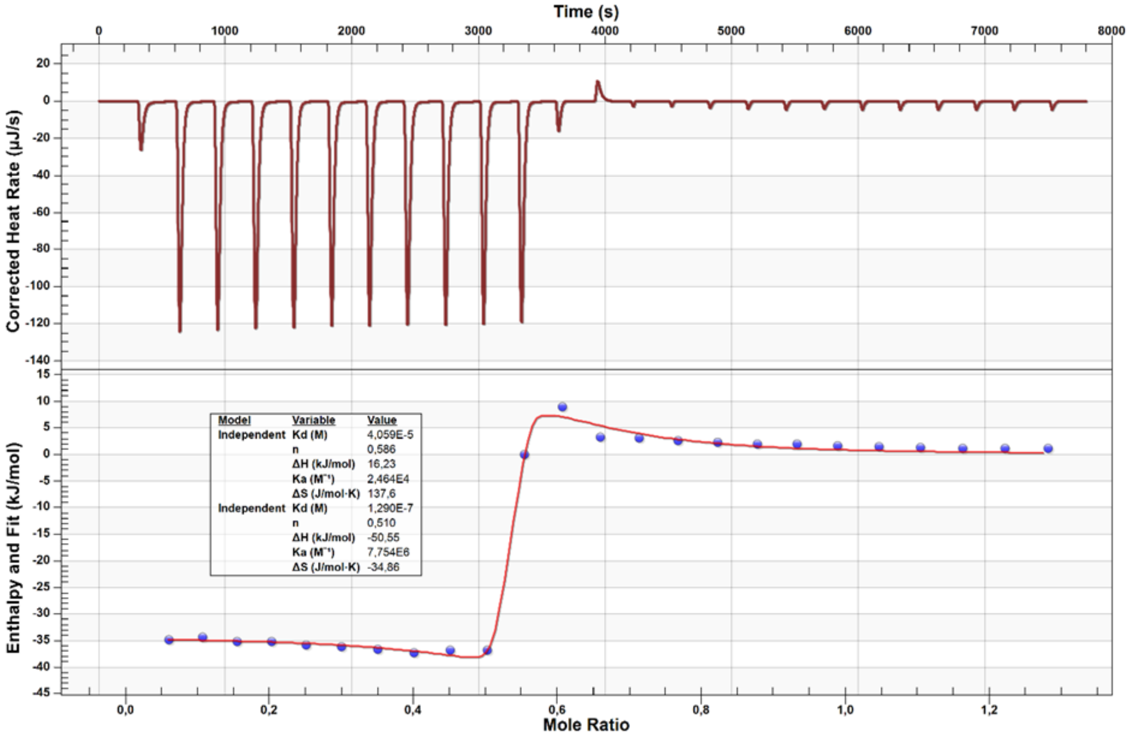

Figure S11: ITC titration data of **Tpy** and **His** (2.39 mM each, in cell) with $Zn(TFMS)_2$ (10.44 mM, in syringe) in $MeOH/CHCl_3$ (2:1) at 303 K.

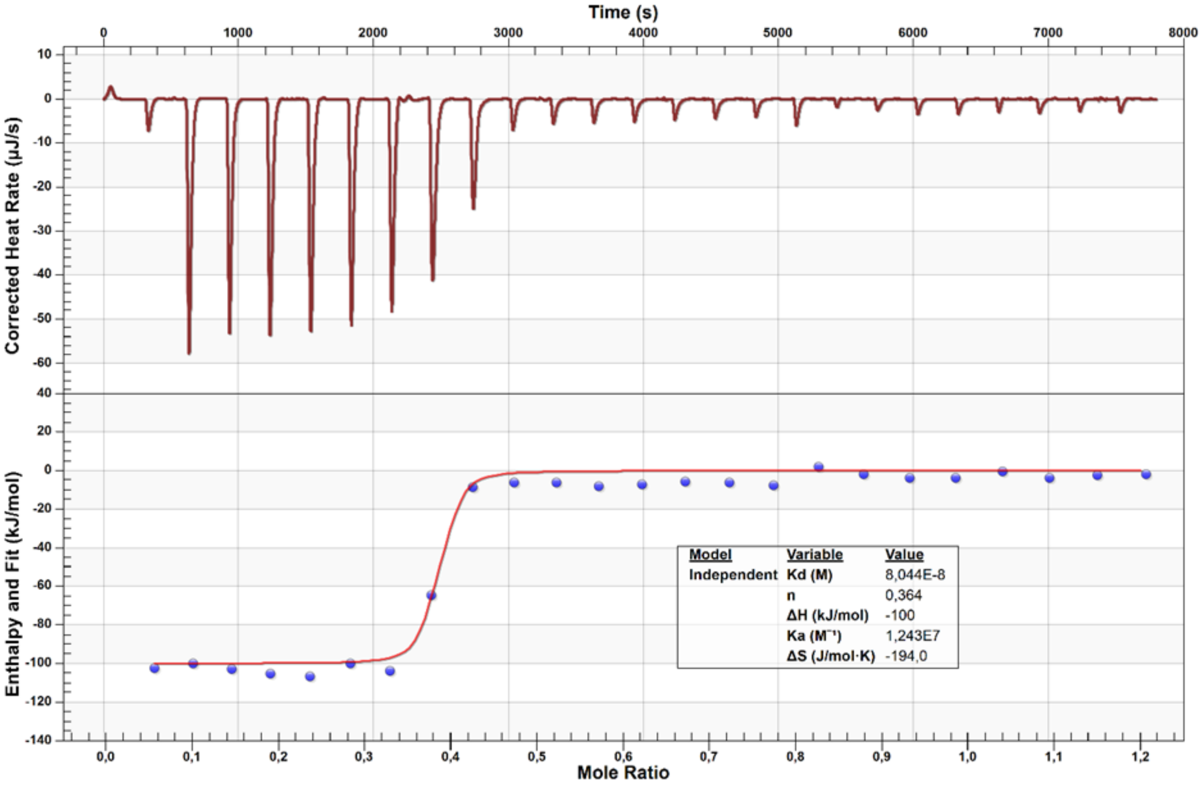

Figure S12: ITC titration data of **Tpy** and **His** (0.35 mM each, in cell) with $NiCl_2 \times 6\ H_2O$ (1.44 mM, in syringe) in $MeOH/CHCl_3$ (2:1) at 303 K.

Synthesis of the model complexes

For the synthesis of the model complexes the ligand (**His** or **Tpy**) was dissolved in chloroform (3 mL) and the respective amount of zinc(II) trifluoromethane sulfonate in case of **[(His)$_2$Zn]$^{2+}$** and **[(Tpy)$_2$Zn]$^{2+}$** or nickel(II) dichloride hexahydrate in case of **[(His)$_3$Ni]$^{2+}$** and **[(Tpy)$_2$Ni]$^{2+}$** was dissolved in methanol (1 mL) and added to the dissolved ligand. The solvent was evaporated and the resulting complex was dried *in vacuo* at 40 °C. A schematic representation is depicted in **Figure S13**. The respective used quantities of all substances are listed in **Table S1**.

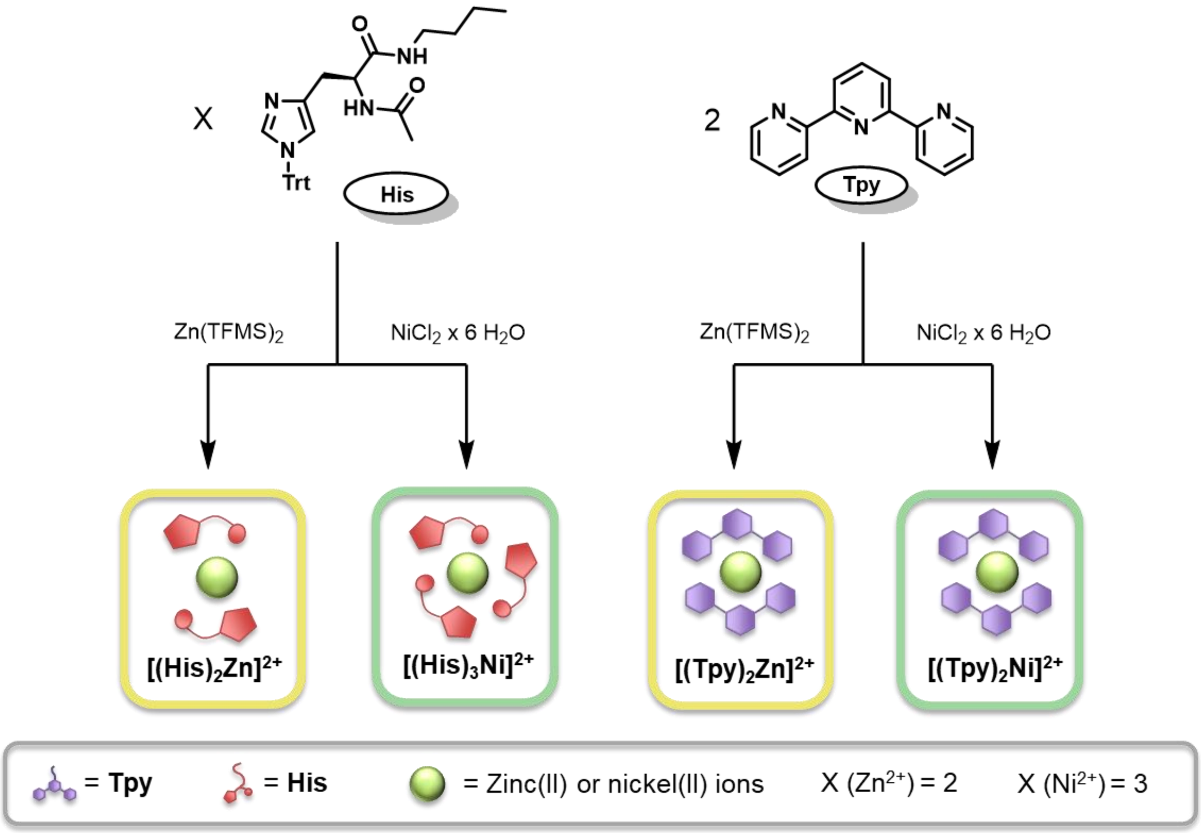

Scheme S3: Schematic representation of the synthesis of the model complexes **[(His)$_2$Zn]$^{2+}$**, **[(Tpy)$_2$Zn]$^{2+}$**, **[(His)$_3$Ni]$^{2+}$** and **[(Tpy)$_2$Ni]$^{2+}$**, respectively.

Table S2: Utilized masses and volumes for the synthesis of the model complexes **[(His)$_2$Zn]$^{2+}$**, **[(Tpy)$_2$Zn]$^{2+}$**, **[(His)$_3$Ni]$^{2+}$** and **[(Tpy)$_2$Ni]$^{2+}$**.

**Metallopolymer**	**Ligand**	**m [mg] (ligand)**	**Metal salt**	**m [mg] (metal salt)**
**[(His)$_2$Zn]$^{2+}$**	**His**	50	Zn(TFMS)$_2$	17
**[(His)$_3$Ni]$^{2+}$**		50	$NiCl_2 \times 6\ H_2O$	8
**[(Tpy)$_2$Zn]$^{2+}$**	**Tpy**	500	Zn(TFMS)$_2$	389
**[(Tpy)$_2$Ni]$^{2+}$**		100	$NiCl_2 \times 6\ H_2O$	51

Proof of the selective complex formation *via* ^{1}H NMR spectroscopy

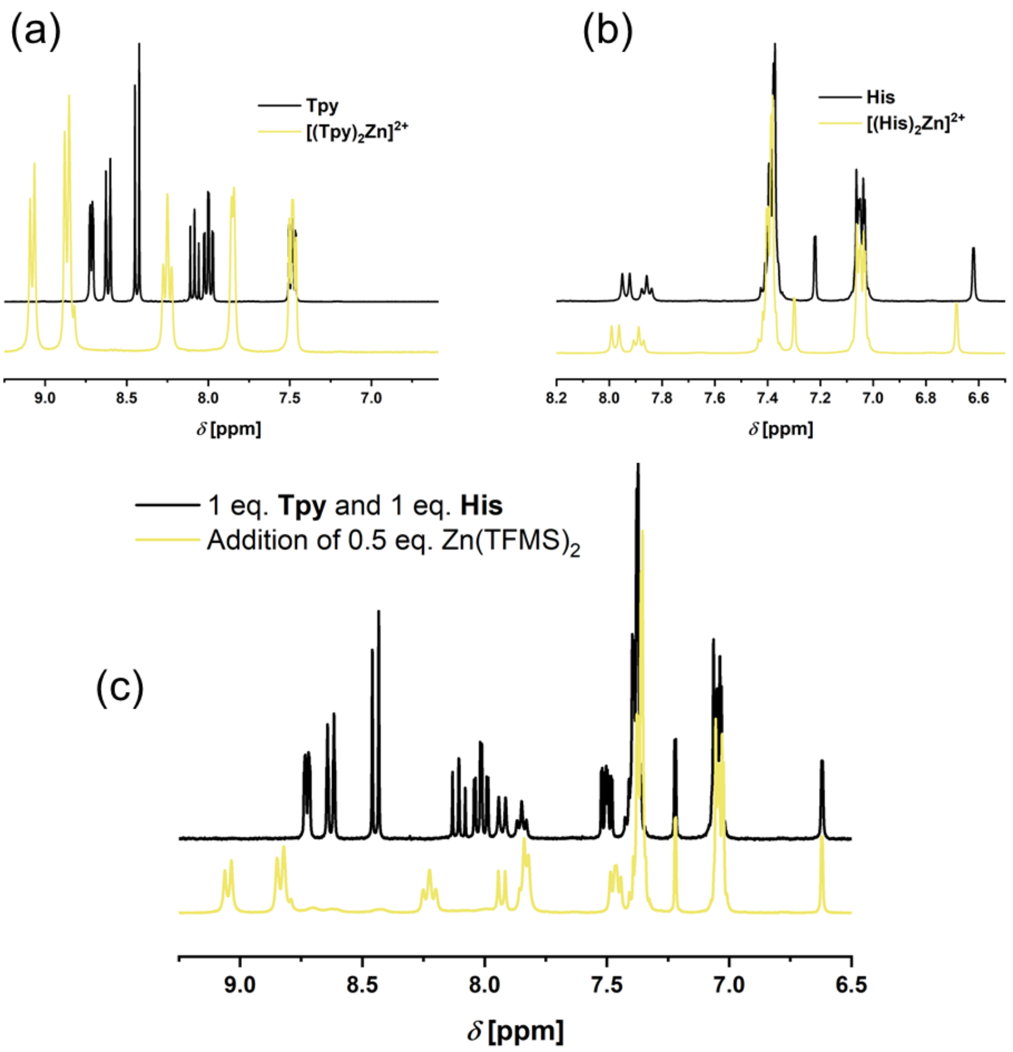

Figure S13: ^{1}H NMR spectra of (a) **Tpy** (black) and **$[(Tpy)_2Zn]^{2+}$** (yellow) (300M Hz, DMSO-d6), (b) **His** (black) and **$[(His)_2Zn]^{2+}$** (yellow) (300 MHz, DMSO-d6) and (c) mixture of one equivalent **Tpy** and **His** (black) after the addition of 0.5 equivalent $Zn(TFMS)_2$ to the mixture leading to the selective formation of **$[(Tpy)_2Zn]^{2+}$**, shift of the terpyridine signals (*e.g.*, at 8.60 to 8.73 ppm, and uncoordinated **His** (yellow), no shift of the histidine signals (*e.g.*, at 6.62 ppm), (300 MHz, DMSO-d6).

Synthesis of the polymers **P1** to **P4** *via* RAFT polymerization

All polymerizations were performed in a 100 mL one-neck-round bottom flask. 2,2'-Azo*bis*(2-methylpropionitrile) (AIBN) as initiator, 2-cyano-2-propyl dodecyl trithiocarbonate (CPDT) as chain transfer agent as well as the monomers N^{α}-methacryloyl-N^{τ}-tritylhistidine butyl amide (**His-MA**), 6-(2,2':6'2''-terpyridin-4'-yloxy)-hexyl methacrylate (**Tpy-MA**) and butyl methacrylate (BMA) were added into the flask. Subsequently, the required amount of toluene was added to reach the desired concentration of 2 M. The [M] to [CPDT] ratio was 125/1, the ratio of [CPDT] to [AIBN] 4/1. The respective used quantities of all substances are listed in **Table S2**. The solution was purged with nitrogen for 1 h. Afterwards, the mixture was stirred for 17 h in a preheated oil bath at 70 °C. The crude product was purified by dialysis (MWCO: 3500 g/mol, THF). The solvent was changed two times each day for three days. The ^{1}H NMR spectra of the polymers **P1** to **P4** are displayed in **Figure S14**. The results of the elemental analysis and the DSC and TGA measurements are summarized in **Table S3**.

Table S3: Utilized masses and volumes for the copolymerization of the polymers **P1** to **P4**.

**Polymer**	**Monomer**	**m [g] (monomer)**	**m [mg] (AIBN)**	**m [mg] (CPDT)**	**V [mL] (toluene)**
	BMA	5.00			
**P1**	**His-MA**	2.75	13.87	116.75	21.1
	**Tpy-MA**	0.74			
	BMA	12.00			
**P2**	**His-MA**	3.30	31.12	262.50	45.4
	**Tpy-MA**	1.76			
	BMA	4.00			
**P3**	**His-MA**	3.94	10.82	91.07	16.5
	**Tpy-MA**	0.35			
	BMA	5.00			
**P4**	**His-MA**	1.37	12.77	107.51	19.4
	**Tpy-MA**	0.44			

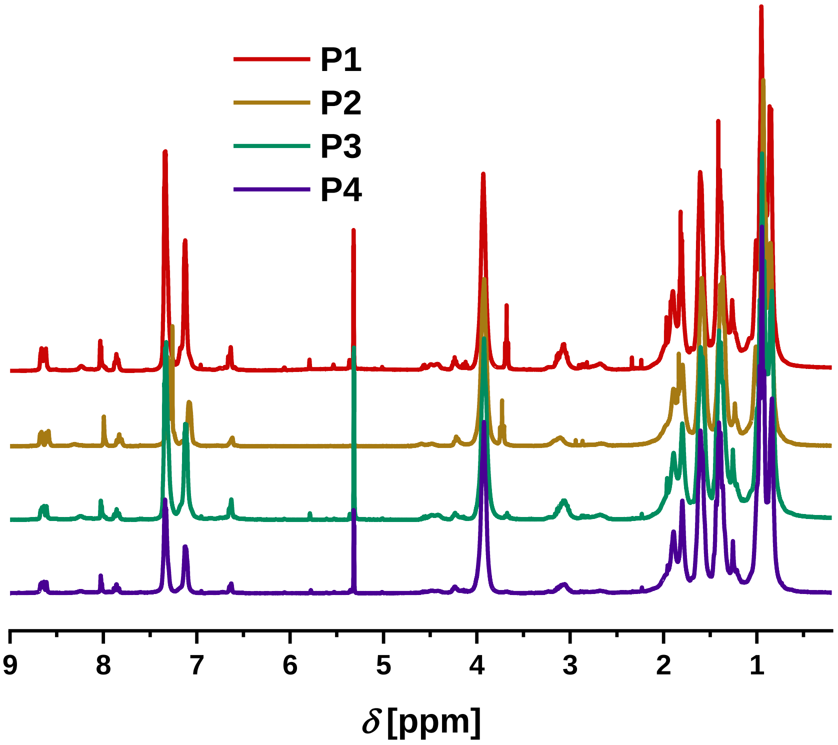

Figure S14: ^{1}H NMR spectra of the polymers **P1** (red), **P2** (yellow), **P3** (green) and **P4** (blue) (300 MHz, CD_2Cl_2).

Table S4: Results of the elemental analysis and the DSC as well as TGA investigations of the polymers **P1** to **P4**.

Polymer	Found in elemental analysis [%]				T_g [°C]	T_d [°C]	
	C	H	N	S		Fast heating [(a)]	Slow heating [(b)]
**P1**	70.38	8.71	4.34	0.28	34	250	199
**P2**	69.31	9.16	2.86	-	28	263	250
**P3**	69.95	8.85	3.88	0.30	34	252	198
**P4**	67.40	8.90	2.67	0.32	25	246	184

[(a)] Heating rate: 20 K min^{-1}; [(b)] Heating rate: 5 K min^{-1}.

Size exclusion chromatography (SEC)

Size exclusion chromatography measurements were performed on the following setup: Shimadzu with CBM-20A (system controller), DGU-14A (degasser), LC-20AD (pump), SIL-20AHT (auto sampler), CTO-10AC vp (oven), SPD-20A (UV detector), RID-10A (RI detector), PSS SDV guard/1000 Å/1,000,000 Å (5 µm particle size) chloroform/isopropanol/triethyl-amine [94/2/4] with 1 mL/ min at 40 °C, poly(methyl methacrylate) (standard).

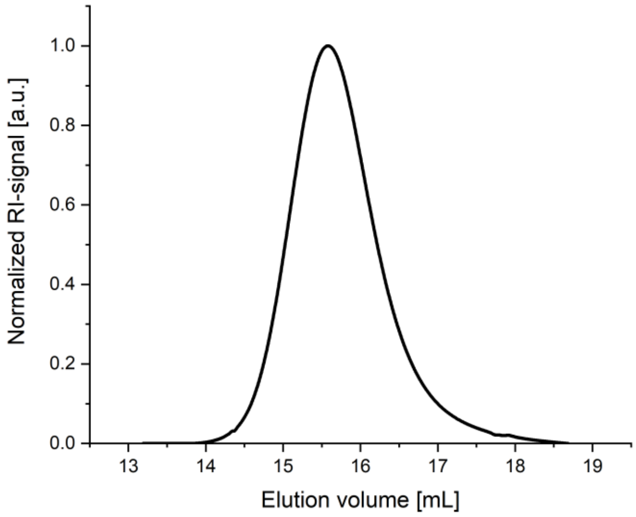

Figure S15: SEC curve of polymer **P1** (eluent: Chloroform/*iso*-propanol/triethylamine [94/2/4]).

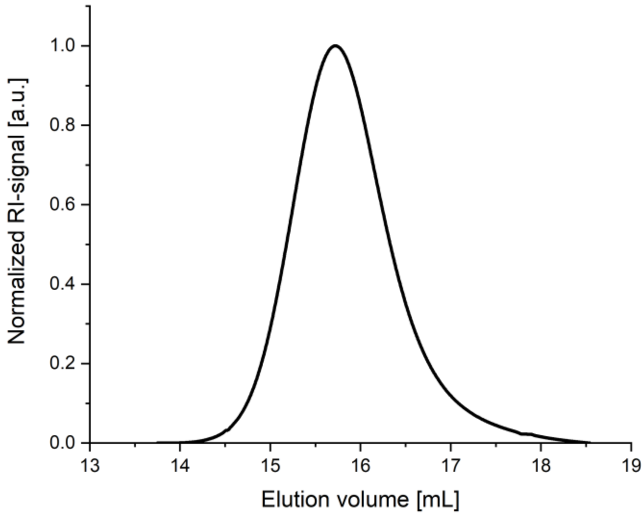

Figure S16: SEC curve of polymer **P2** (eluent: Chloroform/*iso*-propanol/triethylamine [94/2/4]).

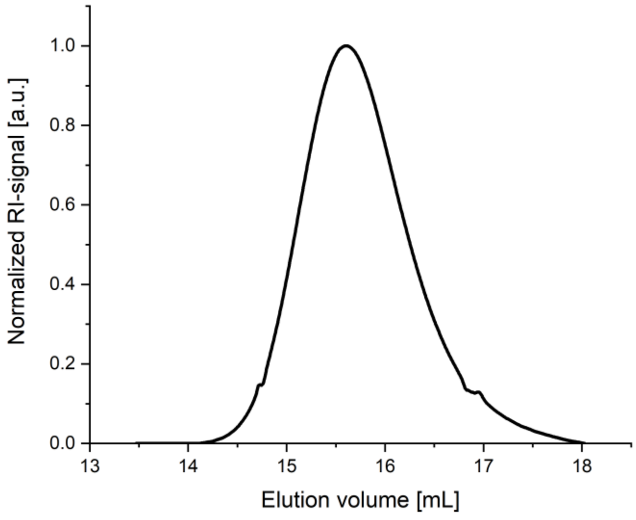

Figure S17: SEC curve of polymer **P3** (eluent: Chloroform/*iso*-propanol/triethylamine [94/2/4]).

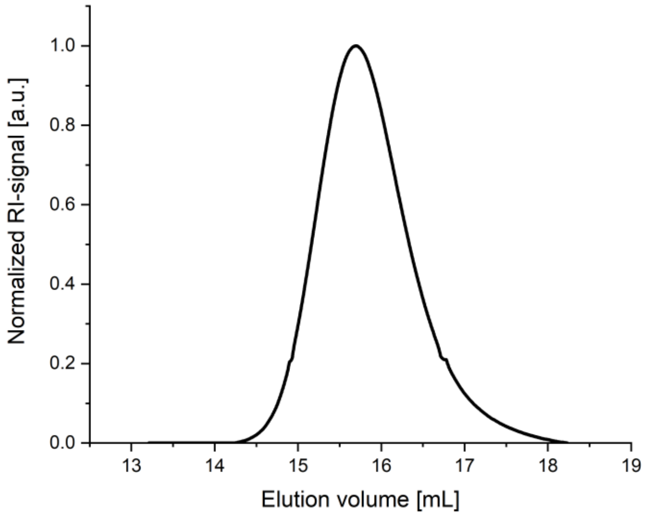

Figure S18: SEC curve of polymer **P4** (eluent: Chloroform/*iso*-propanol/triethylamine [94/2/4]).

Synthesis of the metallopolymer networks

For the synthesis of the metallopolymer networks (**P1-Zn** to **P4-Zn** and **P1-Ni** to **P4-Ni**), the polymers (**P1** to **P4**) were dissolved in chloroform (20 mL). The calculated amount of either zinc(II) trifluoromethane sulfonate or nickel(II) chloride was dissolved in methanol (2 mL) and added to the polymer solution. The solvent was evaporated and the resulting metallopolymer networks was dried *in vacuo* at 40 °C. The respective used quantities of all substances are listed in **Table S4**. The results of the elemental analysis and the DSC and TGA measurements are summarized in **Table S5**.

Table S5: Utilized masses and volumes for the synthesis of the metallopolymer networks **P1-Zn** to **P4-Zn** and **P1-Ni** to **P4-Ni**.

Metallopolymer	Polymer	m [g] (polymer)	Metal salt	m [mg] (metal salt)
**P1-Zn**	**P1**	2.25	Zn(TFMS)$_2$	259
**P1-Ni**		1.55	$NiCl_2 \times 6\ H_2O$	96
**P2-Zn**	**P2**	1.56	Zn(TFMS)$_2$	144
**P2-Ni**		1.53	$NiCl_2 \times 6\ H_2O$	74
**P3-Zn**	**P3**	1.53	Zn(TFMS)$_2$	199
**P3-Ni**		1.51	$NiCl_2 \times 6\ H_2O$	93
**P4-Zn**	**P4**	1.52	Zn(TFMS)$_2$	130
**P4-Ni**		1.58	$NiCl_2 \times 6\ H_2O$	70

Table S6: Results of the elemental analysis and the DSC and TGA investigations of the polymers **P1-Zn** to **P4-Zn** and **P1-Ni** to **P4-Ni**.

Polymer	Found in elemental analysis [%]				T_g [°C]		T_d [°C]	
	C	H	N	S	Range	Turning point	Fast heating [(a)]	Slow heating [(b)]
**P1-Zn**	64.57	7.90	3.99	1.67	65 to 140	93	272	240
**P1-Ni**	67.54	8.28	4.22	-	50 to 135	76	267	225
**P2-Zn**	63.15	8.19	2.62	1.67	55 to 146	71	284	249
**P2-Ni**	65.90	8.69	2.74	-	45 to 127	60	278	225
**P3-Zn**	62.74	7.82	3.52	2.14	62 to 145	95	274	239
**P3-Ni**	67.17	8.52	3.73	0.24	53 to 132	72	266	223
**P3-Zn**	63.78	8.29	2.54	1.55	52 to 148	82	267	234
**P4-Ni**	66.04	8.78	2.63	0.28	52 to 141	73	257	196

[(a)] Heating rate: 20 K min^{-1}; [(b)] Heating rate: 5 K min^{-1}.

Differential scanning calorimetry (DSC)

Differential scanning calorimetry (DSC) was measured on a Netzsch DSC 204 F1 Phoenix instrument (Selb, Germany) under a nitrogen atmosphere with a heating rate of 20 K min^{-1} (first and second heating cycle) and 10 K min^{-1} (third heating cycle).

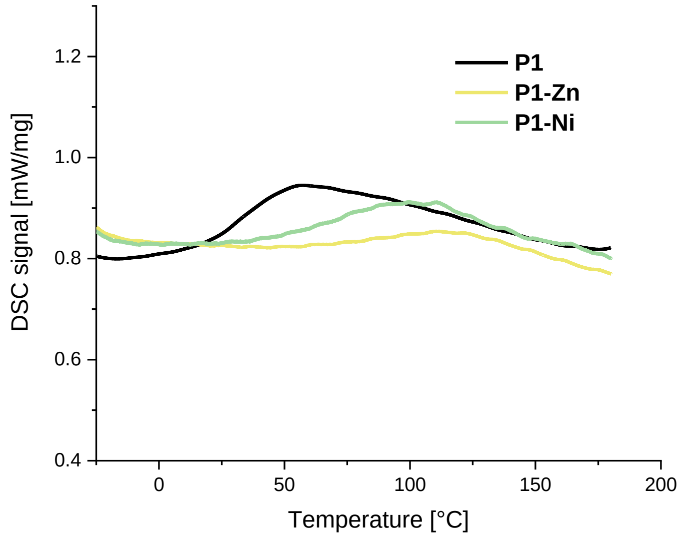

Figure S19: DSC-curves of the second heating cycle of the polymer **P1** (black) and the corresponding metallopolymer networks **P1-Zn** (yellow) and **P1-Ni** (green).

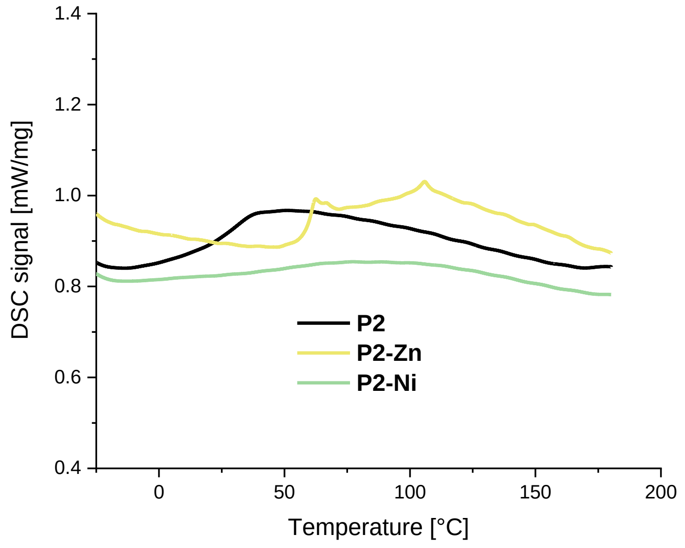

Figure S20: DSC-curves of the second heating cycle of the polymer **P2** (black) and the corresponding metallopolymer networks **P2-Zn** (yellow) and **P2-Ni** (green).

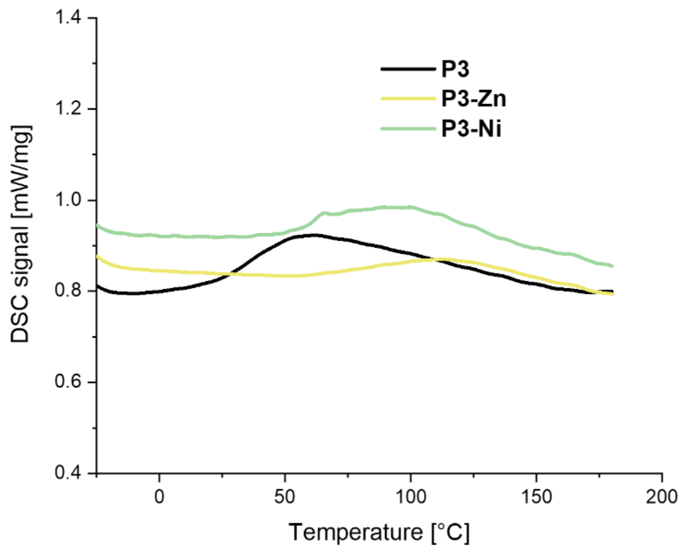

Figure S21: DSC-curves of the second heating cycle of the polymer **P3** (black) and the corresponding metallopolymer networks **P3-Zn** (yellow) and **P3-Ni** (green).

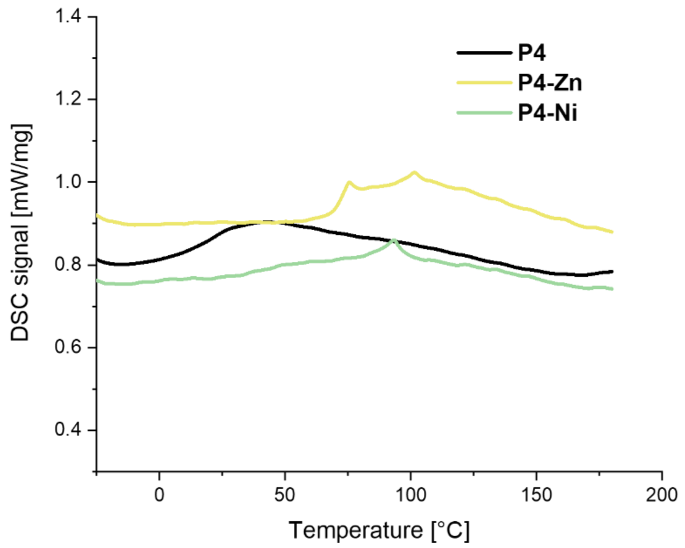

Figure S22: DSC-curves of the second heating cycle of the polymer **P4** (black) and the corresponding metallopolymer networks **P4-Zn** (yellow) and **P4-Ni** (green).

Thermo gravimetric analysis (TGA)

The thermogravimetric analysis (TGA) was carried under normal atmosphere with a heating rate of 20 K min^{-1} and under nitrogen atmosphere with a heating rate of 5 K min^{-1} using a Netzsch TG 209 F1 Iris (Selb, Germany).

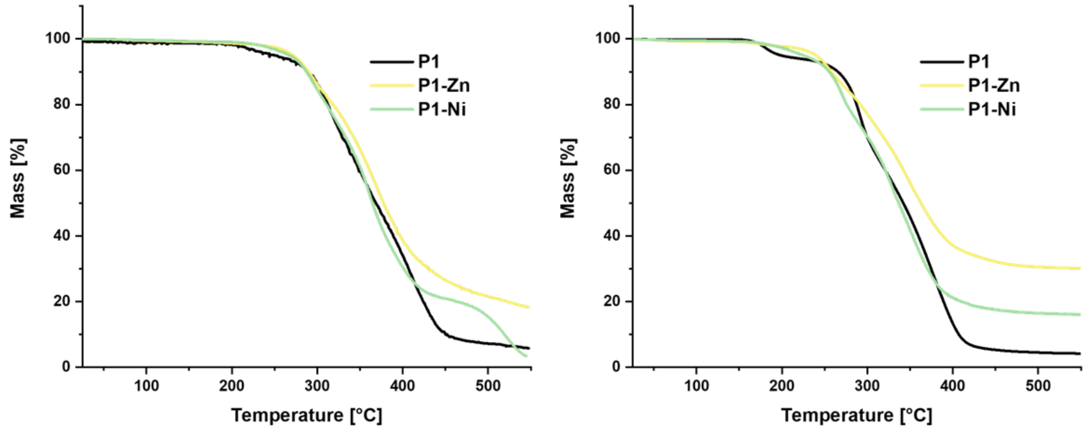

Figure S23: TGA-curves of the polymer **P1** (black) and the corresponding metallopolymer networks **P1-Zn** (yellow) and **P1-Ni** (green) (left: Heating rate = 20 K min^{-1}; right: Heating rate = 5 K min^{-1}).

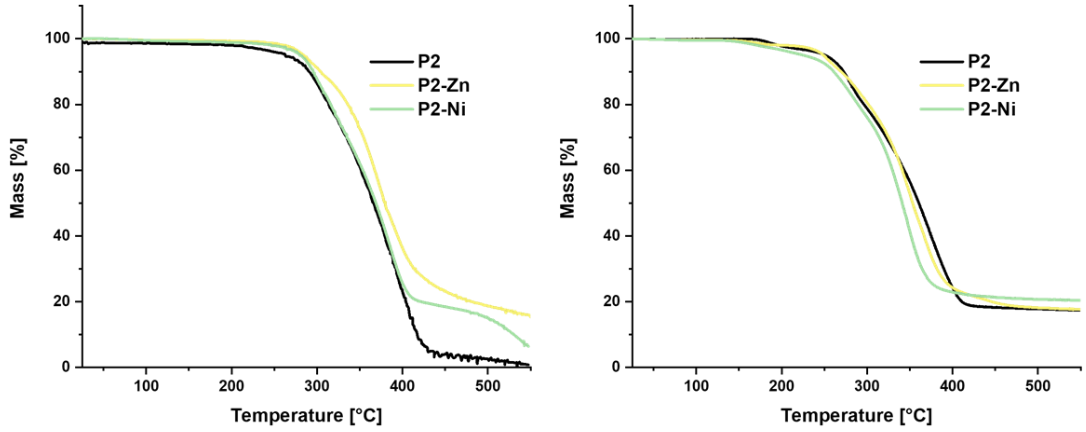

Figure S24: TGA-curves of the polymer **P2** (black) and the corresponding metallopolymer networks **P2-Zn** (yellow) and **P2-Ni** (green) (left: Heating rate = 20 K min^{-1}; right: Heating rate = 5 K min^{-1}).

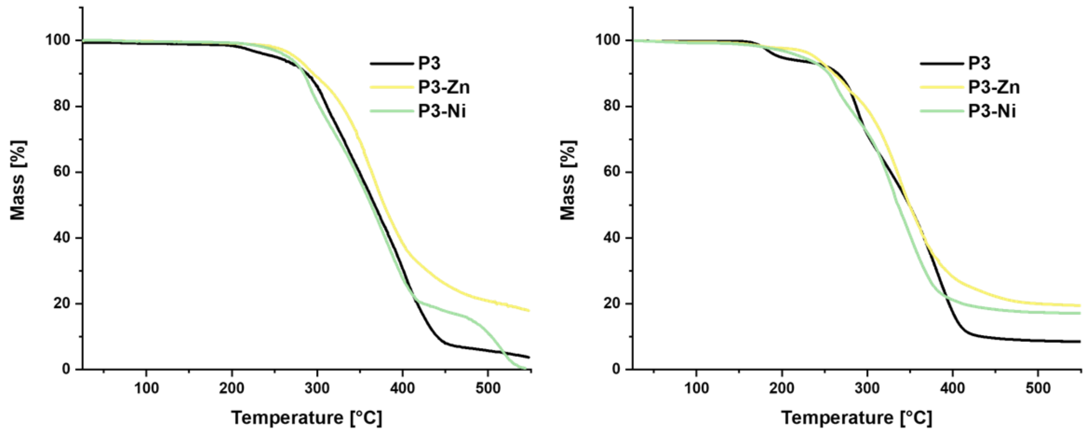

Figure S25: TGA-curves of the polymer **P3** (black) and the corresponding metallopolymer networks **P3-Zn** (yellow) and **P3-Ni** (green) (left: Heating rate = 20 K min^{-1}; right: Heating rate = 5 K min^{-1}).

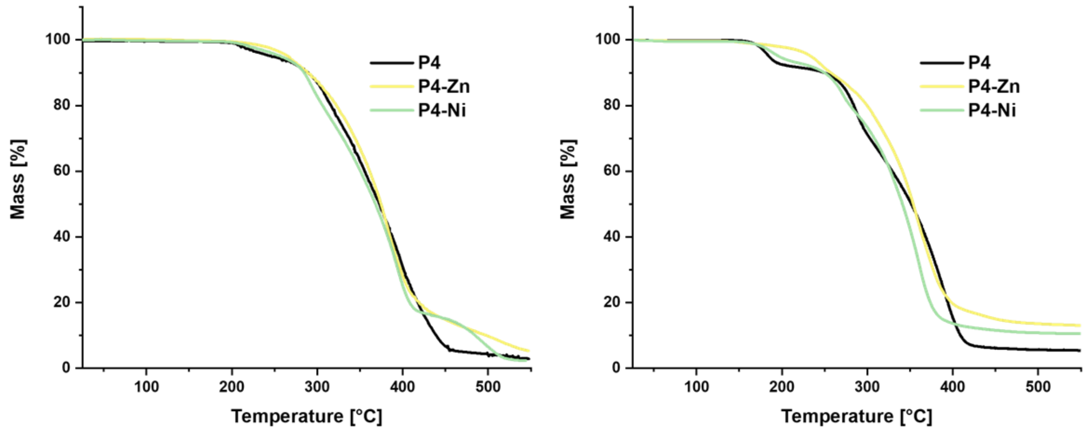

Figure S26: TGA-curves of the polymer **P4** (black) and the corresponding metallopolymer networks **P4-Zn** (yellow) and **P4-Ni** (green) (left: Heating rate = 20 K min^{-1}; right: Heating rate = 5 K min^{-1}).

Cyclo-mechanic tests

For the sample preparation, the metallopolymer networks were pressed at 140 °C at about 1.5 t in rectangular pieces with a special manufactured mold. The cyclo-mechanic-tests were performed using a MCR 301 rheometer from Anton Paar (Graz, Austria) with a convection oven device CTD 450 (temperature range: −150 to 450 °C). The samples were measured with a solid rectangular fixture setup (SRF12-SN13529, Anton Paar (Graz, Austria) in dimensions of approximately 29 × 10 mm (length, width) and a thickness of approx. 1.5 to 3.5 mm, the resulting sample gap was set to 15 mm. For the test, the temperature was set to the respective switching temperature (lowest temperature where it was possible to deform the sample without breaking). Followed by the deformation of the sample (tuning in linear ramp) until the shear stress reached the value, which corresponds to a deformation of about 120° (this value was detected in a prior measurement at the switching temperature). Subsequently, the sample was cooled to 25 °C (10 K min^{-1}) under constant shear stress. Thereafter, the release of the shear stress (to 0 Pa) was performed. For the recovery process, the sample was heated again to the initial temperature (15 K min^{-1}) followed by an annealing step at this temperature. The measurement was repeated three times without interruptions. The software RheoCompassTM V1.24.549-Release 64-bit (Anton Paar, Graz, Austria) was used for the operating of the rheometer as well as for analysis. The data was exported as txt-files and evaluated and processed with OriginPro 2019 (OriginLab Corporation, Northampton, MA, USA).

Table S7: Results of the cyclo-mechanic tests of the metallopolymer networks **P1-Zn/Ni** to **P4-Zn/Ni**.

Metallopolymer	Cycle	γ_p (N-1) [%]	γ_m (N) [%]	γ_u(N) [%]	γ_p (N) [%]
**P1-Zn**	1	0	38.3	38.2	8.63
	2	0	28.5	28.4	4.10
	3	0	26.9	26.8	3.72
	4	0	24.7	24.6	3.19
**P1-Ni**	1	0	22.2	22.1	6.38
	2	0	19.8	19.6	5.62
	3	0	19.0	18.8	6.49
	4	0	14.6	14.5	4.51
**P2-Zn**	1	0	41.8	41.7	5.71
	2	0	35.8	35.7	2.04
	3	0	38.1	38.0	1.79
	4	0	42.4	42.3	2.04
**P2-Ni**	1	0	42.5	42.3	9.53
	2	0	36.2	36.1	5.77
	3	0	34.2	34.1	4.85
	4	0	33.4	33.3	4.52
**P3-Zn**	1	0	27.9	27.8	8.4
	2	0	19.0	18.9	3.6
	3	0	17.6	17.5	3.4
	4	0	16.1	16.0	3.0
**P3-Ni**	1	0	25.5	25.5	9.2
	2	0	22.0	21.9	5.0
	3	0	19.6	19.6	3.8
	4	0	18.0	18.0	3.4
**P4-Zn**	1	0	28.5	28.2	4.0
	2	0	25.9	25.6	2.35
	3	0	25.8	25.5	2.25
	4	0	24.7	24.5	2.28
**P4-Ni**	1	0	25.4	25.0	5.6
	2	0	22.1	21.7	4.7
	3	0	19.8	19.3	4.6
	4	0	17.1	16.7	4.0

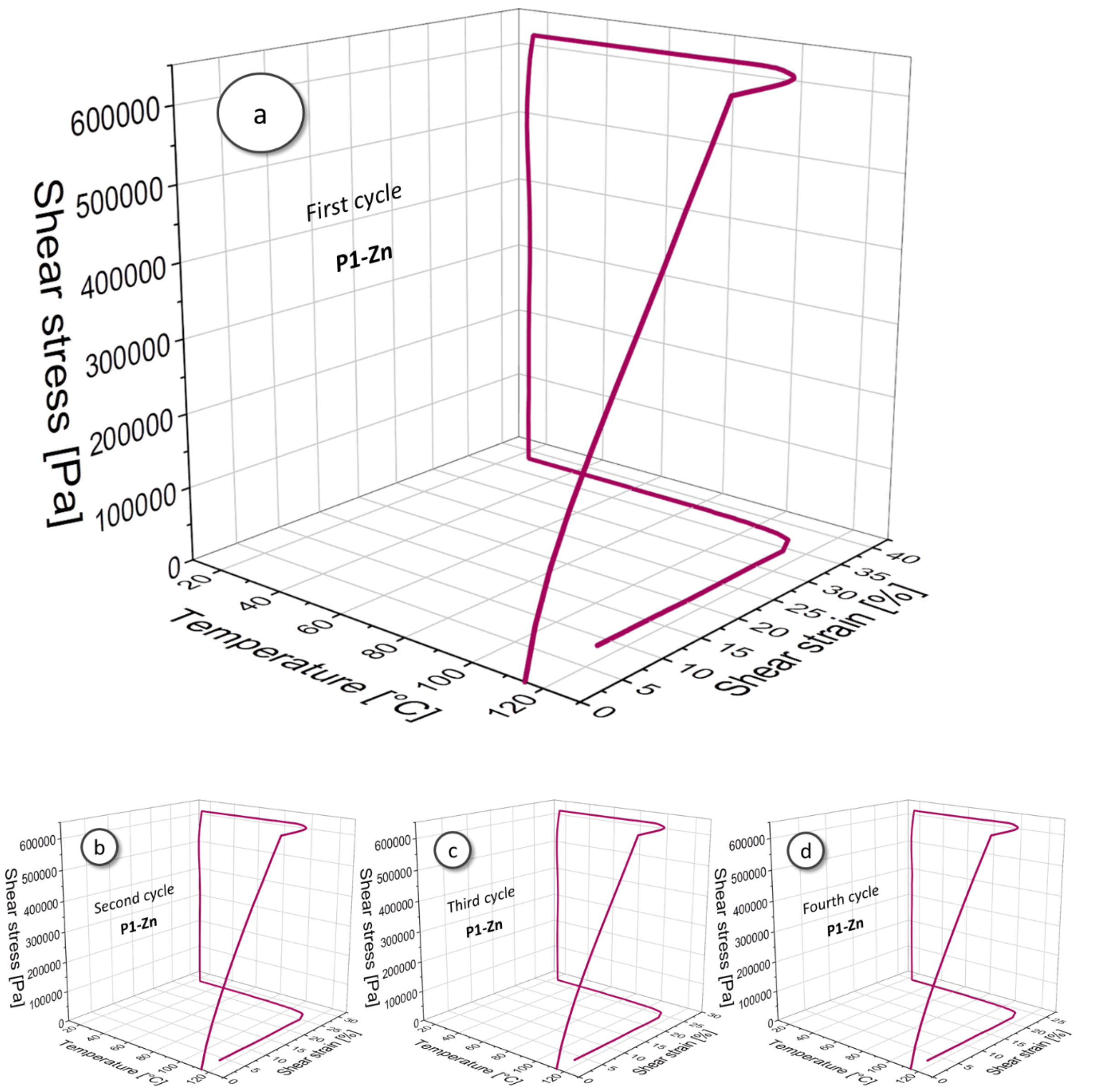

Figure S27: 3D-plots (stress-strain-temperature-diagram) of the cyclo-mechanic test of the metallopolymer network **P1-Zn;** (a) first cycle, (b) second cycle, (c) third cycle and (d) fourth cycle.

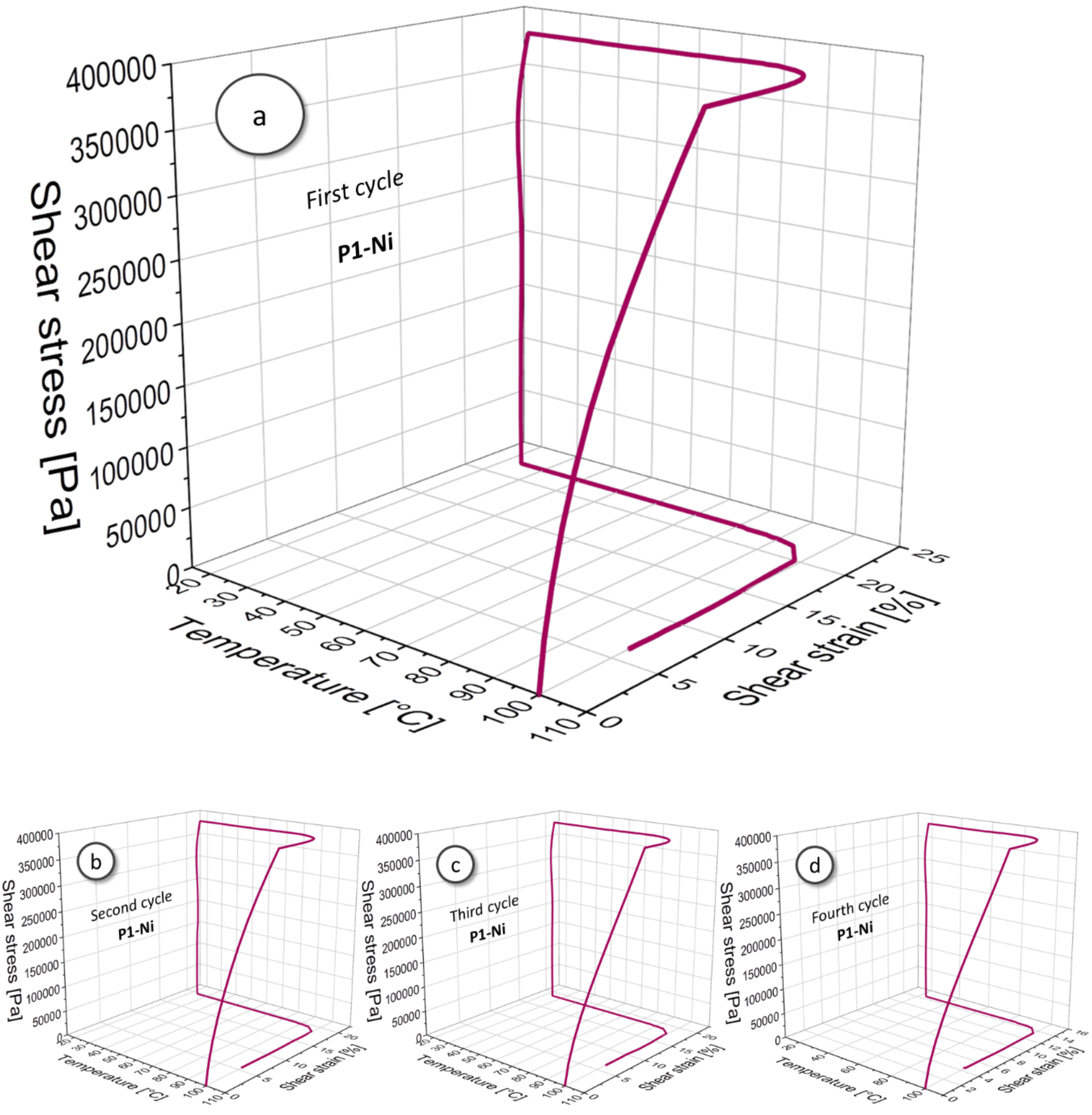

Figure S28: 3D-plots (stress-strain-temperature-diagram) of the cyclo-mechanic test of the metallopolymer network **P1-Ni;** (a) first cycle, (b) second cycle, (c) third cycle and (d) fourth cycle.

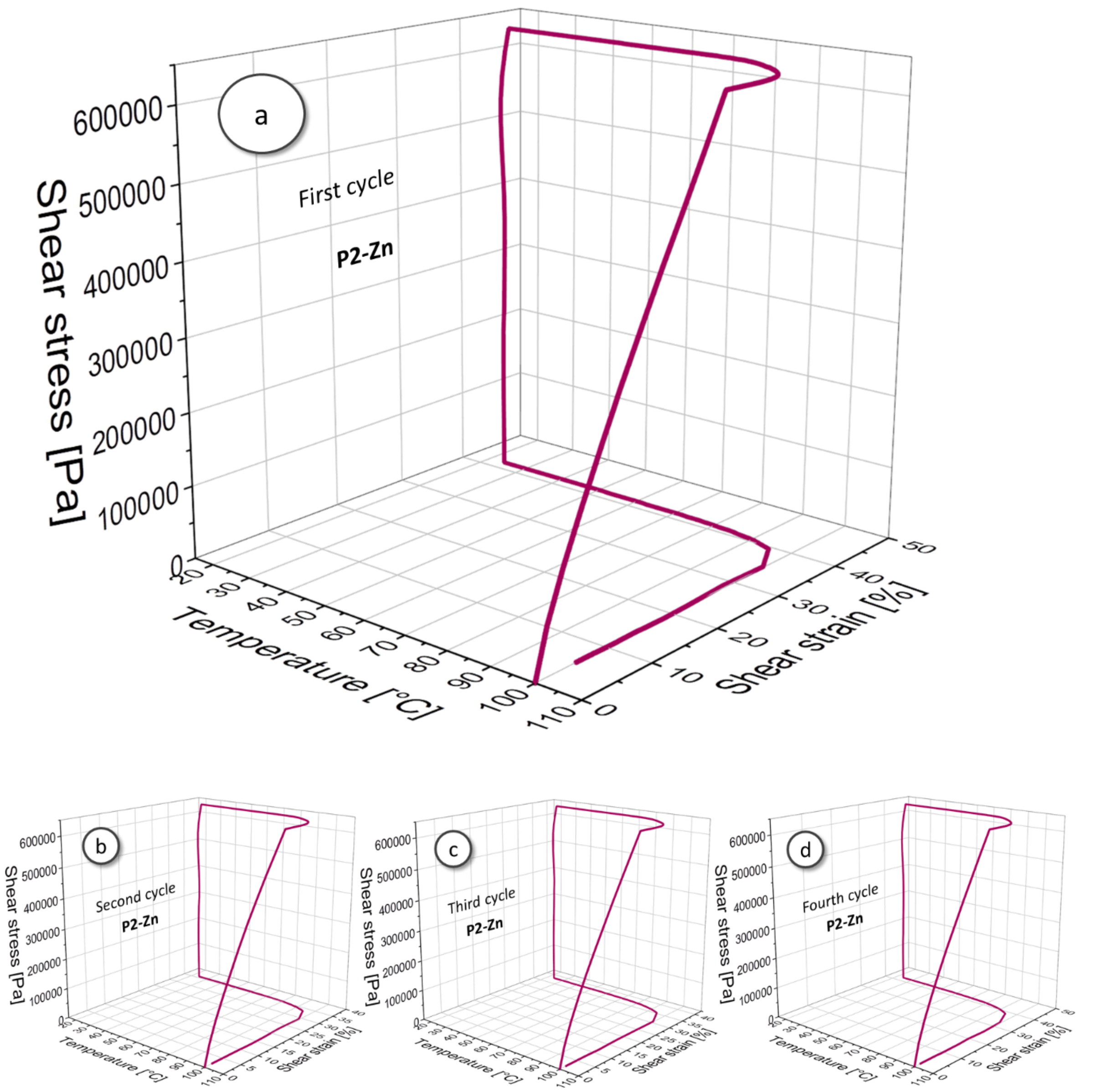

Figure S29: 3D-plots (stress-strain-temperature-diagram) of the cyclo-mechanic test of the metallopolymer network **P2-Zn;** (a) first cycle, (b) second cycle, (c) third cycle and (d) fourth cycle.

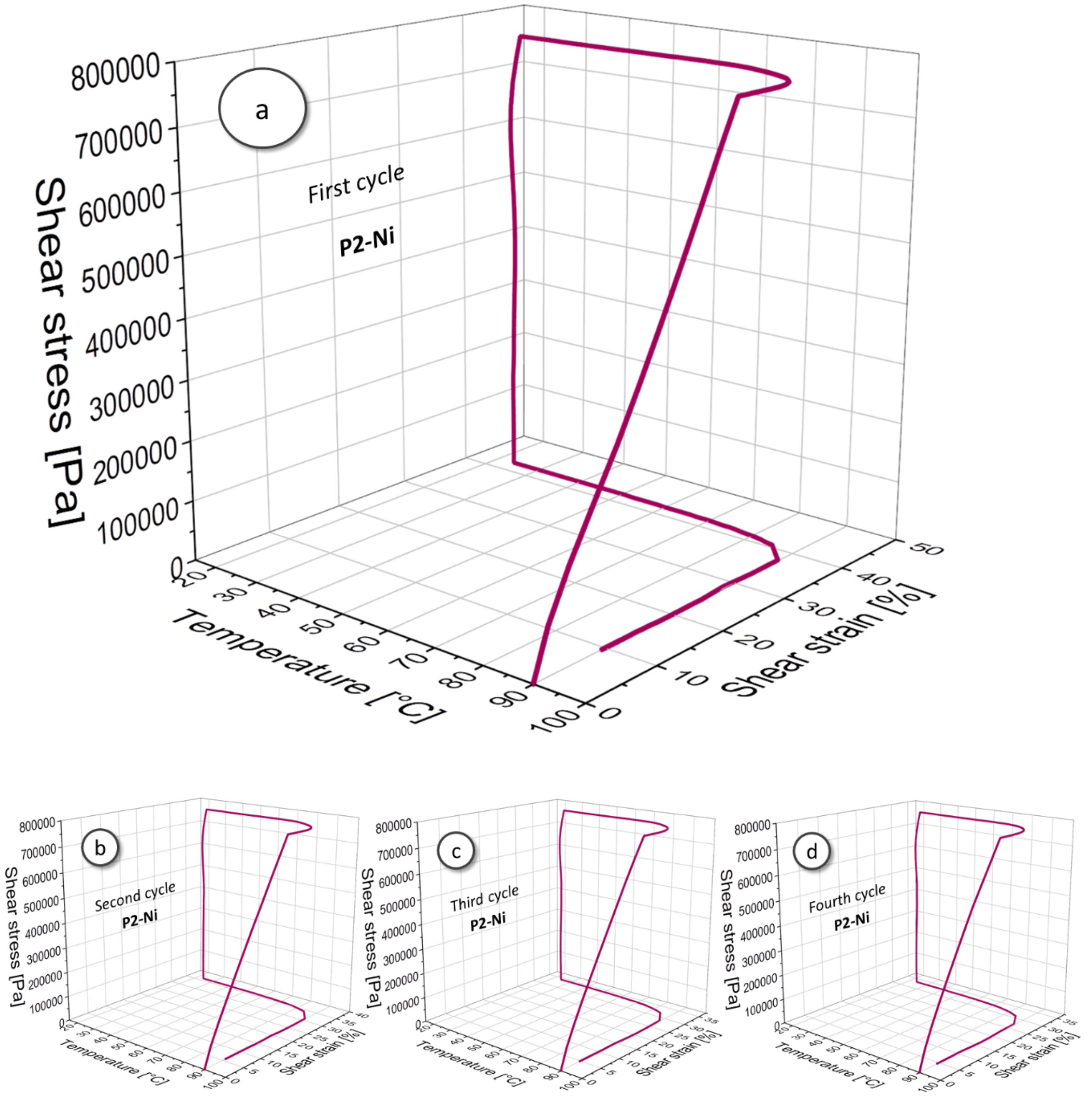

Figure S30: 3D-plots (stress-strain-temperature-diagram) of the cyclo-mechanic test of the metallopolymer network **P2-Ni;** (a) first cycle, (b) second cycle, (c) third cycle and (d) fourth cycle.

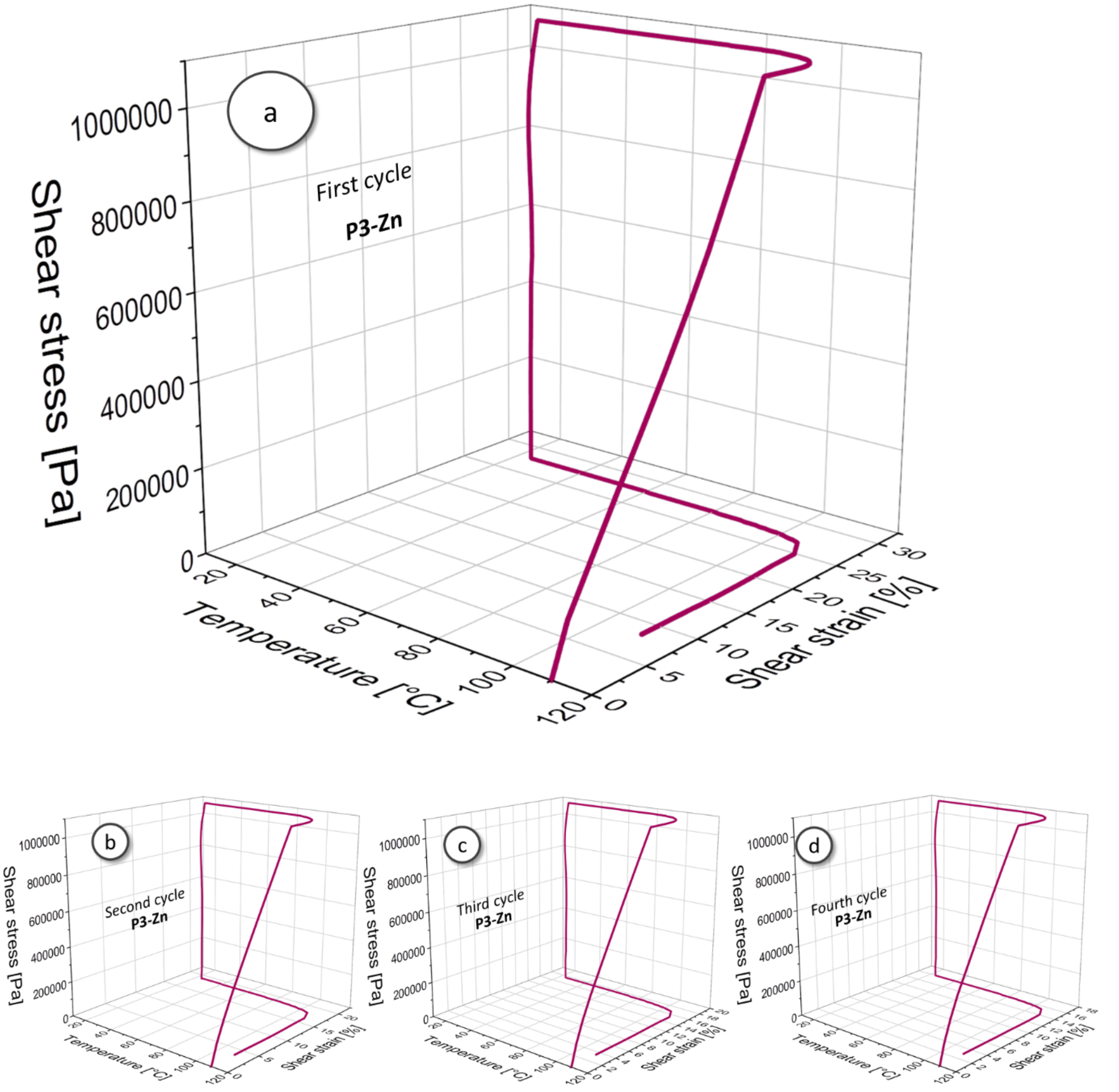

Figure S31: 3D-plots (stress-strain-temperature-diagram) of the cyclo-mechanic test of the metallopolymer network **P3-Zn;** (a) first cycle, (b) second cycle, (c) third cycle and (d) fourth cycle.

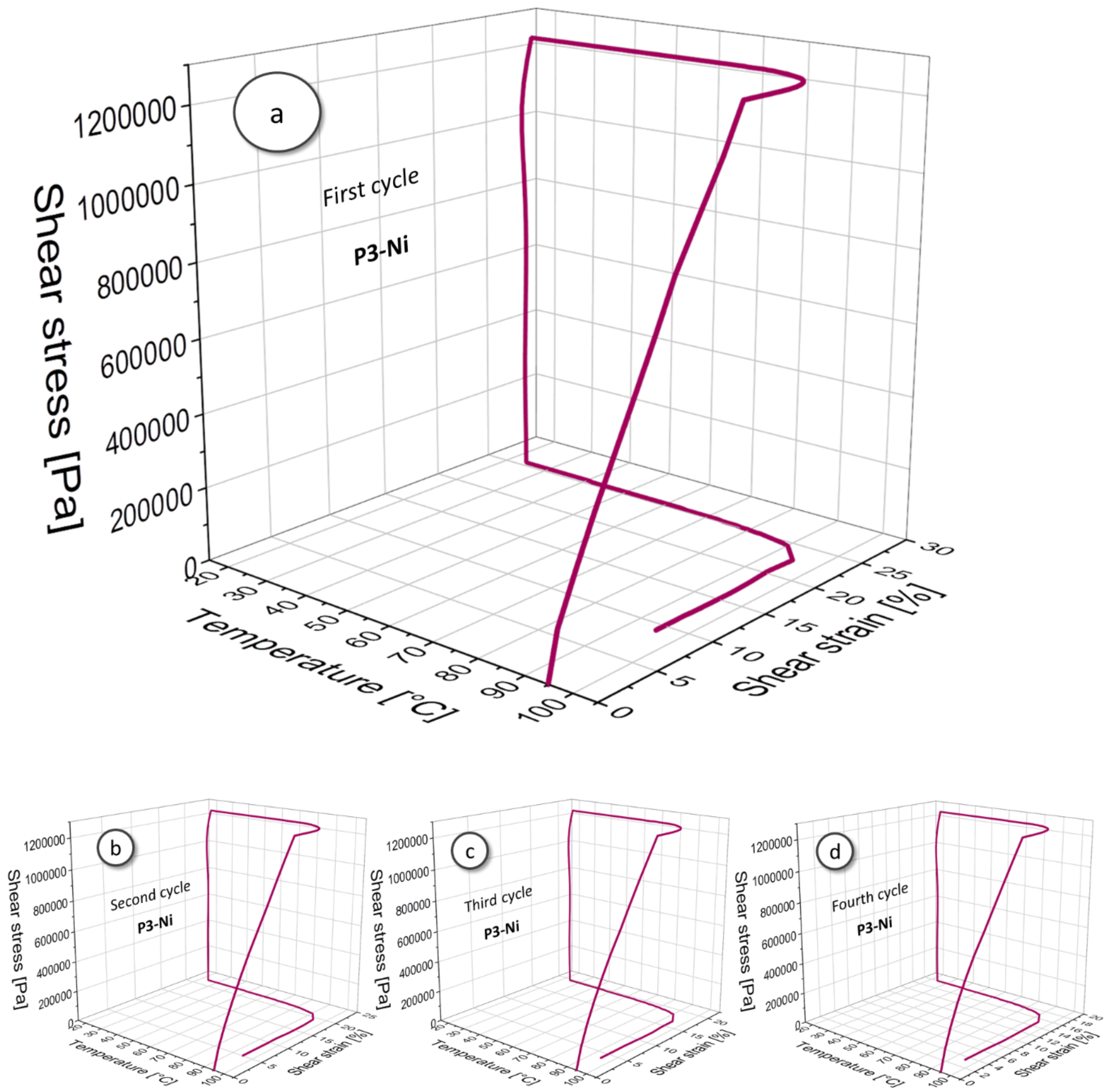

Figure S32: 3D-plots (stress-strain-temperature-diagram) of the cyclo-mechanic test of the metallopolymer network **P3-Ni;** (a) first cycle, (b) second cycle, (c) third cycle and (d) fourth cycle.

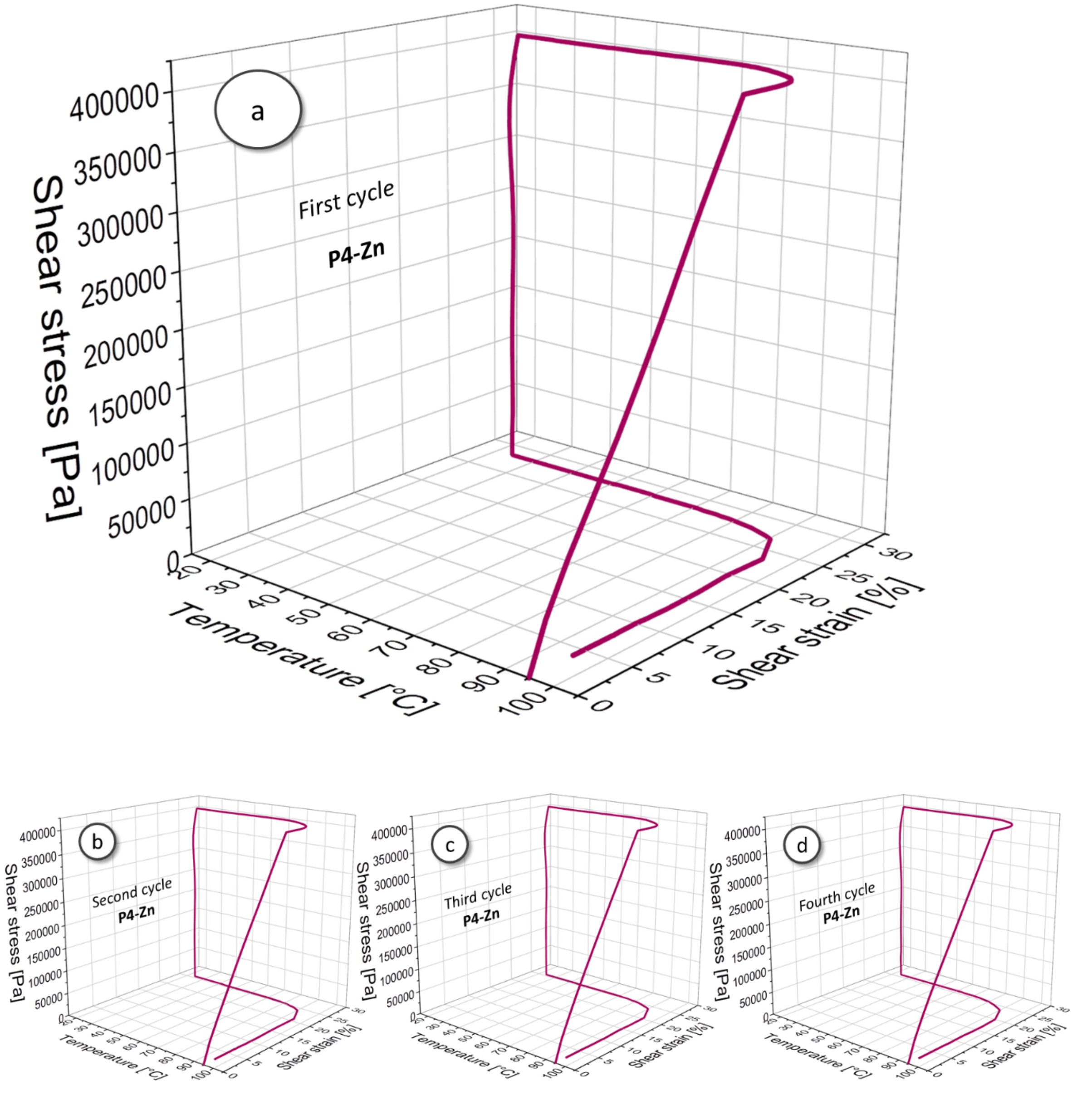

Figure S33: 3D-plots (stress-strain-temperature-diagram) of the cyclo-mechanic test of the metallopolymer network **P4-Zn;** (a) first cycle, (b) second cycle, (c) third cycle and (d) fourth cycle.

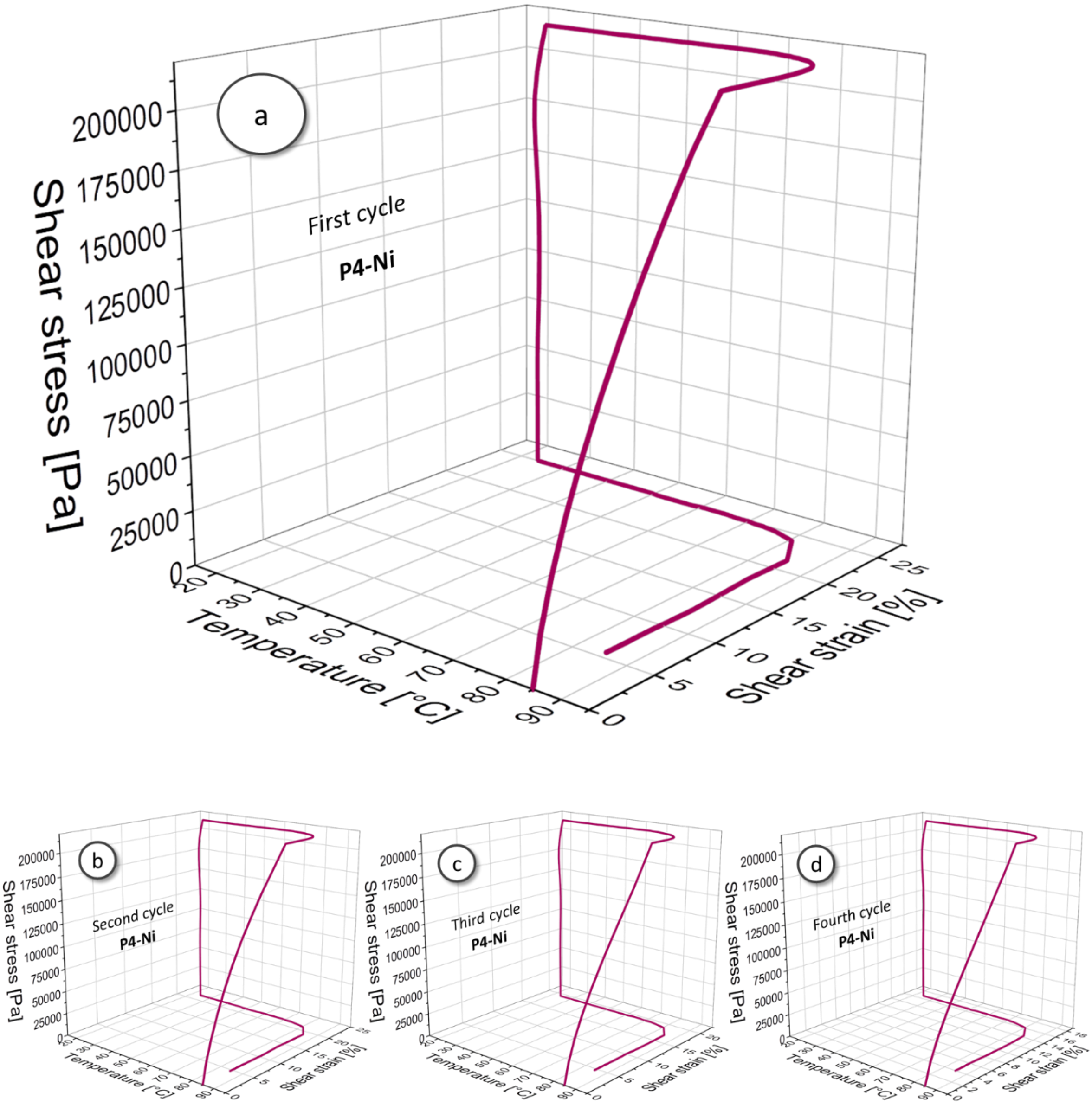

Figure S34: 3D-plots (stress-strain-temperature-diagram) of the cyclo-mechanic test of the metallopolymer network **P4-Ni;** (a) first cycle, (b) second cycle, (c) third cycle and (d) fourth cycle.

Comparison of the R_r depending on the temperature (exemplarily for **P2-Ni**)

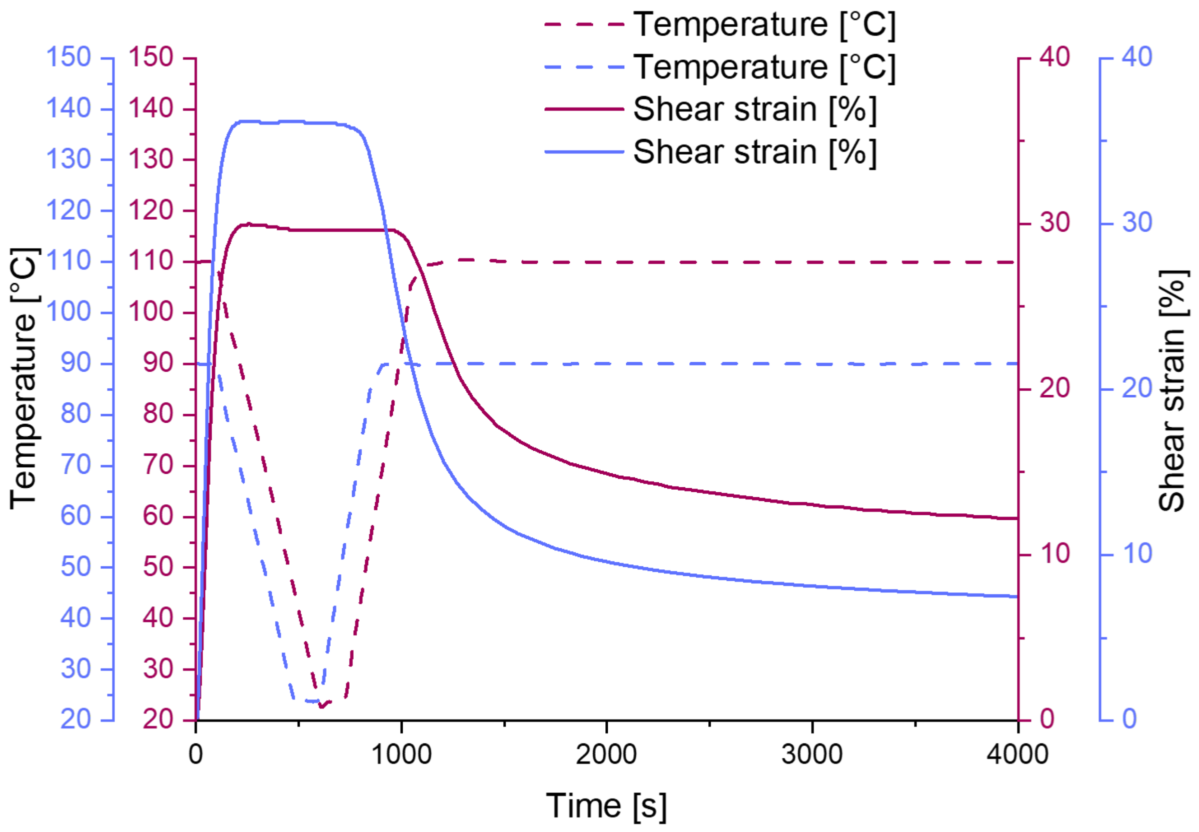

Figure S35: Zoom of the 2D-plot of the cyclo-mechanic test of the metallopolymer network **P2-Ni** at different temperatures**;** (dark-red) 110 °C, (blue) 90 °C. Showing the influence of the ideal temperature on the strain recovery rate of the metallopolymer network.

Raman-spectroscopic measurements

All Raman-spectroscopic measurements were performed on a Multispec Fourier-transform Raman-Spectrometer (Bruker Corporation, Billerica, Massachusetts, United States of America) in the range between 100 and 4000 cm^{-1} with a spectral resolution of 4 cm^{-1}. The Raman excitation light at 1064 nm was provided by a Nd:YAG laser (Klastech DeniCAFC-LC-3/40, Dortmund, Germany). The laser power at the sample had to be varied between 50 and 1000 mW depending on the photothermal stability of the respective sample. To improve the signal-to-noise ratio the number of accumulated scans was varied accordingly. The respective data for each sample can be found in **Table S7**.

Figure S2a and b shows the Raman spectra of the model ligands and their respective complexes with Zn^{2+} and Ni^{2+}. From the model complexes, it is possible to identify characteristic changes of the Raman spectrum upon coordination: For **His,** the broad bands around 1352 and 1435 cm^{-1} reveal shifts to higher wavenumbers, while the band at 1566 cm^{-1} vanishes and the band at 1580 cm^{-1} increases in intensity. This can be interpreted as a shift of the band at 1566 cm^{-1} to higher wavenumbers, so that it merges with the higher energy band. By employing Density-Functional-Theory (DFT) calculations, the bands at 1352 and 1435 cm^{-1} could be assigned to the C-N-stretching vibrations of the imidazole moiety. The band at 1566 cm^{-1} contains strong contributions of the C=C stretching vibration in the imidazole ring (see the SI for detailed band assignment, **Table S9** and **S8**). These shifts are indicative of the typical geometrical changes, which occur upon coordination of a metal ion to histidine. [3]

For **Tpy** the changes in the Raman-spectrum are far more drastic compared to **His**: The sharp bands at 996 (δC-C, δC-N) and 1320 cm^{-1} (νC-C, bridging), shift ca. 30 respectively 20 cm^{-1} to higher wavenumbers, a result of the geometry changes to shorter bond lengths during complexation. [4] Furthermore, more complex changes in the region between 1570 and 1610 cm^{-1} can be observed, which is composed of the C-C/C-N stretching vibrations of the **Tpy** rings. These changes are caused by the conformational change of the **Tpy** from all-*trans*- to all-*cis*-configuration. [4]

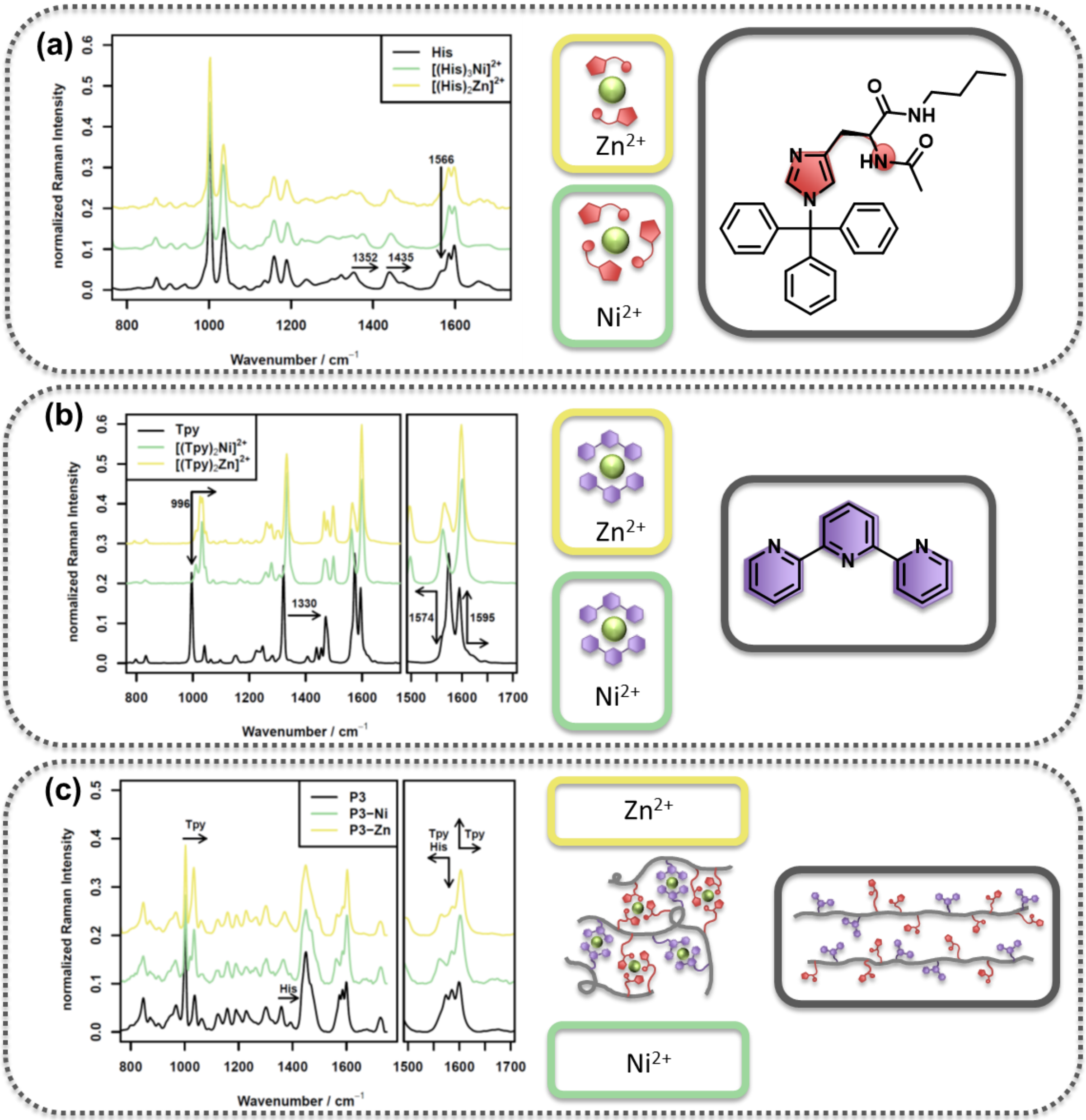

Figure S36. Raman-spectra of (a) **His** (black) and the model complexes **$[(His)_2Zn]^{2+}$** (red), **$[(His)_3Ni]^{2+}$** (blue); (b) **Tpy** (black) and the model complexes **$[(Tpy)_2Zn]^{2+}$** (red), **$[(Tpy)_2Ni]^{2+}$** (blue); (c) polymer **P3** (black) and the corresponding metallopolymer networks **P3-Zn** (red), **P3-Ni** (blue).

Table S8: Laser power at sample and number of accumulated scans for the FT-Raman measurements of all samples.

Sample	Laser Power (mW)	Number of Scans	Sample	Laser Power (mW)	Number of Scans
**Tpy-**	200	128	**P2**	1000	128
**$[(Tpy)_2Zn]^{2+}$**	200	128	**P2-Zn**	1000	128
**$[(Tpy)_2Ni]^{2+}$**	50	2048	**P2-Ni**	200	1024
**His**	1000	128	**P3**	1000	128
**$[(His)_2Zn]^{2+}$**	1000	128	**P3-Zn**	1000	128
**$[(His)_3Ni]^{2+}$**	50	2048	**P3-Ni**	200	1024
**P1**	1000	128	**P4**	1000	128
**P1-Zn**	1000	128	**P4-Zn**	1000	128
**P1-Ni**	200	1024	**P4-Ni**	200	1024

For further evaluation the raw Raman data was preprocessed using R 3.6.1[5]: The spectra were restricted to the wavenumber of interest (*e.g.*, 700 to 1750 cm^{-1}) followed by background correction using the SNIP-algorithm (iterations: 50, order: 3, smoothing window: 3) and normalized using euclidean vector norm. [6] Peak finding was achieved by fitting a fmm-spline to the Raman spectra and then numerically determining gradient and hessian of the spline to find local maxima in the function. [7]

Density functional theory calculations

To assign the observed vibrational patterns to the functional groups of the ligands **Tpy** and **His** Density Functional Theory (DFT) calculations were performed on the respective structures. The calculations were performed using Orca (version 4.2) [8, 9] For both structures geometry optimization and frequency calculation were performed using the gradient corrected functional BLYP [10, 11] and the triple-ζ basis-set def2-TVZP combined with empirical dispersion correction using the Becke-Johnson damping scheme. [12-14] To speed up the calculation resolution of identity approximation for the coloumb integrals with the auxiliary basis set def2/J was used. [15, 16] To account for the neglection of the vibrational

anharmonicity in the frequency calculation the calculated frequencies were scaled with the empirically determined factor 0.998. [17]

Band assignments for **Tpy** and **His**

Table S9: Observed band positions and intensities alongside DFT calculated positions and assignment for **Tpy** in the wavenumber range between 700 and 1750 cm^{-1}.

Position	Rel. intensity	Calculated position	Assignment
725	0.13	724	δ(C-C), in plane
798	0.04	796	δ(C-C), out-of plane
833	0.07	828	ν_{as}(C-C), briding<br>δ(C-C). in-plane
996	0.79	984	δ(C-C), scissoring
1041	0.16	1034	δ(C-C), scissoring
1064	0.03	1057	δ(C-C), scissoring
1097	0.03	1089 / 1094	δ(C-H), in plane
1153	0.07	1148	δ(C-H), in plane
1226	0.12	1227	ν_{as}(C-C), ring
1248	0.16	1251	ν_{as}(C-C), ring
1283	0.07	1281	δ(C-H), in plane
1321	0.89	1304	ν_{s}(C-C), bridging<br>δ(C-C). in-plane
1407	0.06	1388	ν_{s}(N-C), central
1439	0.14	1427	ν_{as}(N-C), ring
1456	0.14	1439	ν_{as}(C-C), ring
1471	0.43	1458	ν_{as}(C-C), ring
1559	sh	1540	ν_{s}(C-C), outer ring<br>ν_{as}(C-C), central ring
1574	1.00	1552	ν(C-C), ring

Position	Rel. intensity	Calculated position	Assignment
1595	0.69	1564	ν_s(C-C), central ring<br>νa_s(C-C), outer ring

Table S10: Observed band positions and intensities alongside DFT calculated positions and assignment for **His** in the wavenumber range between 700 and 1750 cm^{-1}.

Position	Relative intensity	Calculated position	Assignment
752	0.02	745	δ(C-H), out-of-plane
872	0.08	860	δ(C-H), phenyl rings<br>ν(N-C), imidazole-trityl
905	0.04	893	δ(C-C), δ(C-H) butyl chain
942	0.04	930	δ(C-H), out-of-plane, phenyl rings
1003	1.00	1001	δ(C-C), phenyl rings
1036	0.39	1028	δ(C-C), phenyl rings
1086	0.02	1087	δ(C-H)
1118	sh	1110	ν(N-C), imidazole-trityl, isopropyl amide
1136	0.06	1119	ν(N-C), butyl amide
1159	0.21	1141	ν_s(C-C), trityl carbon
1189	0.19	1177	ν_{as}(C-C), trityl carbon
1238	0.06	1240	ν(N-C), imidazole<br>δ(N-H), amide
1301	0.07	1307	δ(C-H), twisting, butyl chain
1322	0.09	1314	δ(C-H), δ(C-C)

Position	Relative intensity	Calculated position	Assignment
1353	0.11	1323	ν(C-N), imidazole
1435	0.11	1432	ν(C-N), imidazole<br>ν(C-H), aliphatic chains
1469	0.04	1467	ν(C-H), scissoring, isopropyl
		1522	ν(C=C), imidazole
1562	0.12	1564	ν(C=C), phenyl
		1569	ν(C=C), phenyl
1585	0.23	1583	ν(C=C), phenyl
1598	0.28	1591	ν(C=C), phenyl
1657	0.05	1649	ν(C=O), amide

Raman-spectra of polymer(s) (networks)

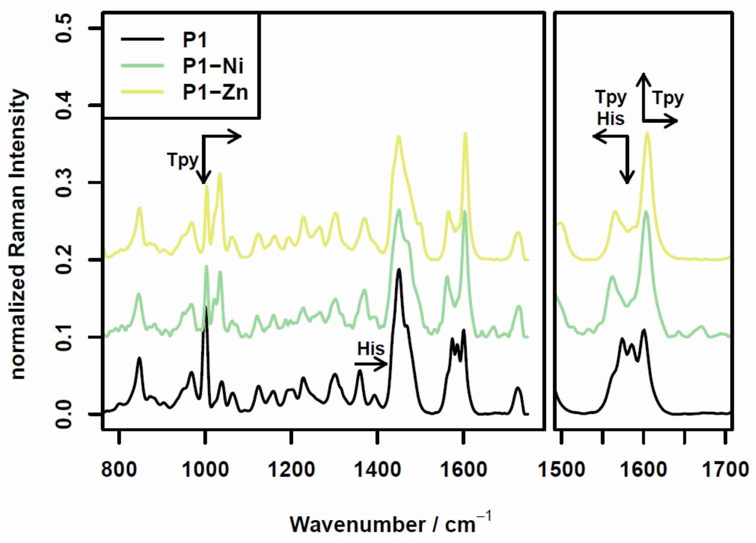

,

Figure S37: Raman spectra of polymer **P1** (black, bottom) and the corresponding metallopolymer networks **P1-Ni** (green, middle) and **P1-Zn** (yellow, top) in the wavenumber range between 800 and 1700 cm^{-1}. The arrows indicate changes in the Raman spectrum upon coordination of a metal ion and the respective coordination site that these changes originate from.

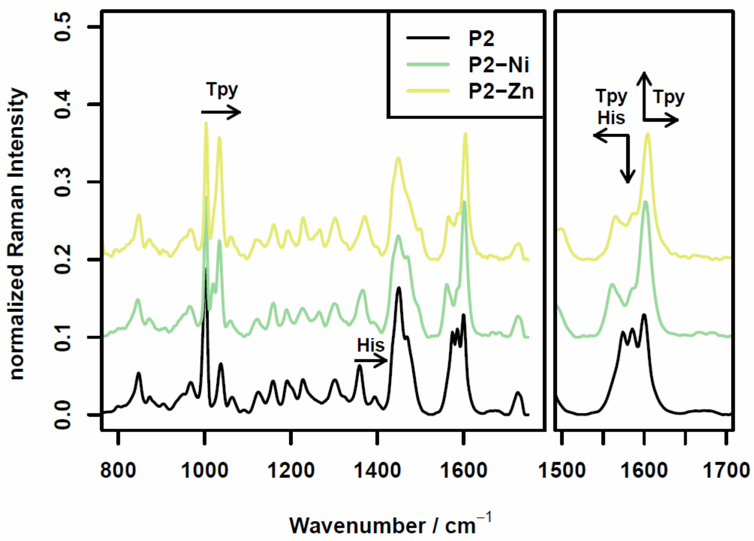

Figure S38: Raman spectra of polymer **P2** (black, bottom) and the corresponding metallopolymer networks **P2-Ni** (green, middle) and **P2-Zn** (yellow, top) in the wavenumber range between 800 and 1700 cm^{-1}. The arrows indicate changes in the Raman spectrum upon coordination of a metal ion and the respective coordination site that these changes originate from.

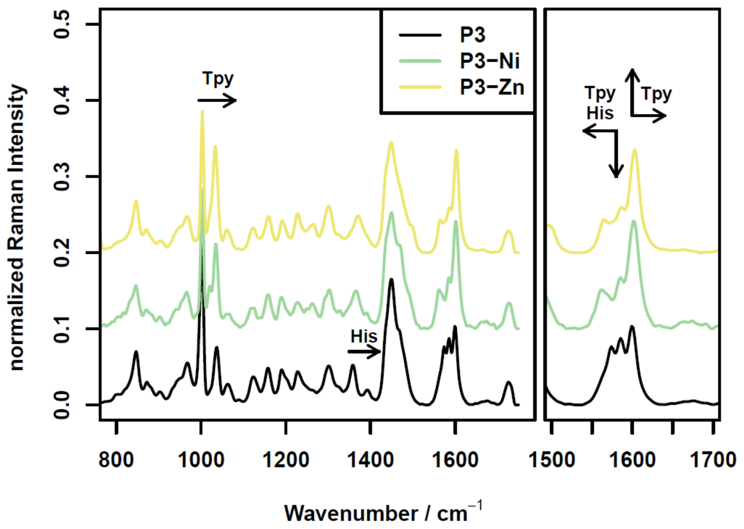

Figure S39: Raman spectra of polymer **P3** (black, bottom) and the corresponding metallopolymer networks **P3-Ni** (green, middle) and **P3-Zn** (yellow, top) in the wavenumber range between 800 and 1700 cm^{-1}. The arrows indicate changes in the Raman spectrum upon coordination of a metal ion and the respective coordination site that these changes originate from.

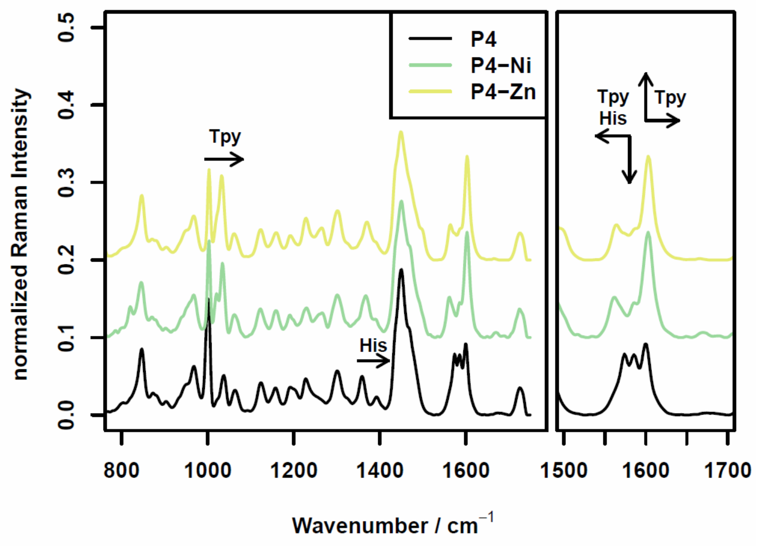

Figure S40: Raman spectra of polymer **P4** (black, bottom) and the corresponding metallopolymer networks **P4-Ni** (green, middle) and **P4-Zn** (yellow, top) in the wavenumber range between 800 and 1700 cm^{-1}. The arrows indicate changes in the Raman spectrum upon coordination of a metal ion and the respective coordination site that these changes originate from.

Additional photo series of the shape-memory test

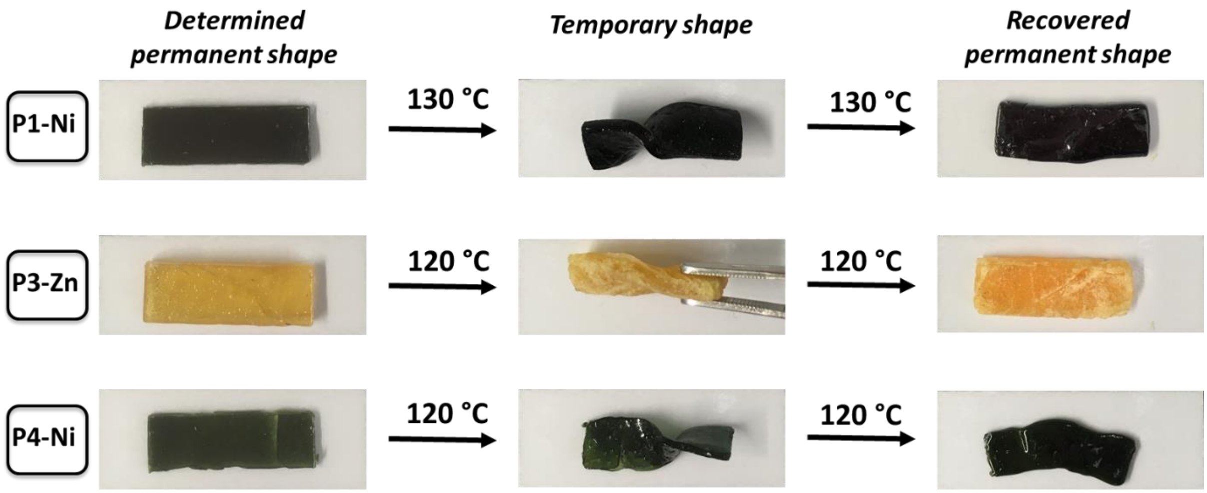

Figure S41: Photo series of the manual performed shape memory test for the metallopolymer networks **P1-Ni**, **P3-Zn** and **P4-Ni**.

References

[1] M. Enke, F. Jehle, S. Bode, J. Vitz, M. J. Harrington, M. D. Hager, U. S. Schubert, *Macromol. Chem. Phys.* **2017**, *218*, 1600458.

[2] S. Bode, L. Zedler, F. H. Schacher, B. Dietzek, M. Schmitt, J. Popp, M. D. Hager, U. S. Schubert, *Adv. Mater.* **2013**, *25*, 1634-1638.

[3] K. Hasegawa, T.-a. Ono, T. Noguchi, *J. Phys. Chem. B* **2000**, *104*, 4253-4265.

[4] M. Presselt, B. Dietzek, M. Schmitt, J. Popp, A. Winter, M. Chiper, C. Friebe, U. S. Schubert, *J. Phys. Chem. C* **2008**, *112*, 18651-18660.

[5] R-Core-Team, *Vienna, Austria* **2019**.

[6] C. G. Ryan, E. Clayton, W. L. Griffin, S. H. Sie, D. R. Cousens, *Nucl. Instrum. Methods Phys. Res.* **1988**, *34*, 396-402.

[7] P. G. a. R. Varadhan, **2019**.

[8] F. Neese, *WIREs Computational Molecular Science* **2018**, *8*, e1327.

[9] F. Neese, *WIREs Computational Molecular Science* **2012**, *2*, 73-78.

[10] A. D. Becke, *Phys. Rev. A* **1988**, *38*, 3098-3100.

[11] C. Lee, W. Yang, R. G. Parr, *Phys. Rev. B* **1988**, *37*, 785-789.

[12] F. Weigend, R. Ahlrichs, *Phys. Chem. Chem. Phys.* **2005**, *7*, 3297-3305.

[13] S. Grimme, S. Ehrlich, L. Goerigk, *J. Comput. Chem.* **2011**, *32*, 1456-1465.

[14] S. Grimme, J. Antony, S. Ehrlich, H. Krieg, *J. Chem. Phys.* **2010**, *132*, 154104.

[15] K. Eichkorn, O. Treutler, H. Öhm, M. Häser, R. Ahlrichs, *Chem. Phys. Lett.* **1995**, *240*, 283-290.

[16] F. Weigend, *Phys. Chem. Chem. Phys.* **2006**, *8*, 1057-1065.

[17] R. D. Johnson III, **2019**.
